# Assessment of health state utilities in dermatology: an experimental time trade-off value set for the dermatology life quality index

**DOI:** 10.1186/s12955-022-01995-x

**Published:** 2022-06-03

**Authors:** Gábor Ruzsa, Fanni Rencz, Valentin Brodszky

**Affiliations:** 1 Department of Statistics, Corvinus University of Budapest, 8 Fővám tér, 1093 Budapest, Hungary; 2 Doctoral School of Psychology, Institute of Psychology, Eötvös Loránd University, 46 Izabella u., 1064 Budapest, Hungary; 3 Department of Health Economics, Corvinus University of Budapest, 8 Fővám tér, 1093 Budapest, Hungary

**Keywords:** Dermatology life quality index, Health-related quality of life, Utility, Time trade-off, Value set

## Abstract

**Background:**

Dermatology Life Quality Index (DLQI) scores are used in many countries as access and reimbursement criteria for costly dermatological treatments. In this study we examined how time trade-off (TTO) utility valuations made by individuals from the general population are related to combinations of DLQI severity levels characterizing dermatologically relevant health states, with the ultimate purpose of developing a value set for the DLQI.

**Methods:**

We used data from an online cross-sectional survey conducted in Hungary in 2020 (*n* = 842 after sample exclusions). Respondents were assigned to one of 18 random blocks and were asked to provide 10-year TTO valuations for the corresponding five hypothetical health states. To analyze the relationship between DLQI severity levels and utility valuations, we estimated linear, censored, ordinal, and beta regression models, complemented by two-part scalable models accommodating heterogeneity effects in respondents’ valuation scale usage. Successive severity levels (0–3) of each DLQI item were represented by dummy variables. We used cross-validation methods to reduce the initial set of 30 dummy variables and improve model robustness.

**Results:**

Our final, censored linear regression model with 13 dummy variables had *R*^2^ = 0.136, thus accounting for 36.9% of the incremental explanatory power of a maximal (full-information) benchmark model (*R*^2^ = 0.148) over the uni-dimensional model (*R*^2^ = 0.129). Each DLQI item was found to have a negative effect on the valuation of health states, yet this effect was largely heterogeneous across DLQI items, and the relative contribution of distinctive severity levels also varied substantially. Overall, we found that the social/interpersonal consequences of skin conditions (in the areas of social and leisure activities, work and school, close personal relationships, and sexuality) had roughly twice as large disutility impact as the physical/practical aspects.

**Conclusions:**

We have developed an experimental value set for the DLQI, which could prospectively be used for quantifying the quality-adjusted life years impact of dermatological treatments and serve as a basis for cost-effectiveness analyses. We suggest that, after validation of our main results through confirmatory studies, population-specific DLQI value sets could be developed and used for conducting cost-effectiveness analyses and developing financing guidelines in dermatological care.

**Supplementary Information:**

The online version contains supplementary material available at 10.1186/s12955-022-01995-x.

## Background

Health-related quality of life (HRQoL) assessments in dermatology and other medical areas have wide applicability including clinical trials, patient registries, diagnostic criteria, and treatment decisions [1]. HRQoL is also a widely used outcome measure for estimating quality-adjusted life years (QALYs) in cost-effectiveness analyses concerning medical interventions. For this latter purpose HRQoL is required to be measured on a utility scale.

Utility values are typically derived using some cardinal elicitation technique (standard gamble method, time trade-off valuation, etc.), and they are measured on a scale anchored at 1 (perfect health) and 0 (death) [2]. Another way of obtaining utility valuations is with the use of generic multi-attribute utility instruments such as the EQ-5D, the SF-6D, and the Health Utilities Index [3]. Yet, in the area of skin diseases these measures may not capture sufficiently well the full range of important health problems associated with specific dermatological conditions (e.g. itching, skin irritation, and decreased self-confidence have been identified as important aspects of the HRQoL burden associated with many skin diseases but not covered by generic utility instruments [4]).

Specialty- or condition-specific measures take better account of the types and degrees of impairment caused by skin diseases [5]. Among them, the Dermatology Life Quality Index (DLQI) is the most frequently used skin-specific HRQoL measure [6, 7], the validity, reliability, and responsiveness of which has been confirmed by numerous studies across a variety of skin conditions [8]. A shortcoming of the DLQI, however, is that its outcome combinations have hitherto not been valued on a utility scale, and previous studies have reported discrepancies between DLQI scores and utilities assessed by skin disease patients as well as by the general population [9, 10].

Recently there has been extensive research into the development of ‘mapping models’, which aim to predict EQ-5D utility valuations from DLQI scores [11–15]. Yet, the majority of existent mapping models were developed for psoriasis populations, thus they cannot be used reliably with other skin conditions. Furthermore, mapping models have been reported to perform poorly at the lower end of the utility scale due to the relatively small number of patients with severe symptoms [16].

A possible solution to these problems would be to develop a utility value set for the DLQI, which could be used at all severity levels in any dermatological disease area [17, 18]. Similar value sets have been developed for condition-specific HRQoL measures in other disease areas including overactive bladder syndrome, asthma, cancer, and dementia [19]. Such tools provide valuable information for the economic evaluation of treatment options and they facilitate policy decision making in healthcare.

Motivated by these concerns, our research objective was to investigate how the ten items underlying the DLQI relate to individuals’ utility valuation of dermatologically relevant health states as assessed by the time trade-off (TTO) method. Thus, we aimed to develop a statistical model providing estimated TTO utilities for all possible combinations of DLQI severity levels, which could ultimately be used as a societal value set.

## Methods

Our research was based on a cross-sectional sample survey conducted in Hungary in February 2020. We followed the Checklist for Reporting Valuation Studies [20] to describe all important aspects of the study design.

### Data collection

Data were collected through an online survey to which respondents were recruited from the adult general population of Hungary. In as much as relevant to this study, the survey consisted of two parts. The first set of questions were concerned with participants’ demographic characteristics including their gender, age, marital status, level of education, employment status, place of residence, and geographic region. In the second part respondents were asked to provide utility valuations for hypothetical health states.

Participants were recruited from an online panel consisting of over 150 thousand individuals. We hired a survey company to select the sample by way of non-probabilistic quota sampling, aiming to ensure representativeness in terms of the main demographic characteristics. Informed consent was obtained from each participant prior to starting the survey.

The online questionnaire was completed by 2459 individuals, 458 of whom were excluded due to quota requirements. Data provided by the remaining 2001 participants were used as input for the statistical analyses.

### Valuation of health states

Participants were asked to provide utility valuations for five hypothetical, dermatologically relevant health states. These were described in terms of their skin disease-related negative impacts on life quality, corresponding to specific combinations of DLQI severity levels.

#### Dermatology life quality index

The DLQI [6] is a 10-item self-completion questionnaire designed to assess the negative impact of skin diseases concerning distinctive aspects of HRQoL, belonging to one of six broader categories: symptoms and feelings, daily activities, social and leisure activities, work and school, personal relationships, and treatment (“Appendix A.1”). The response categories on each item and the corresponding scores are as follows: ‘not at all’ / ‘not relevant’ (0); ‘a little’ (1); ‘a lot’ (2); ‘very much’ (3).

In mathematical terms, the DLQI gives rise to 4^10^≈1 million possible combinations of severity levels. Of these, 73 hypothetical health states were chosen, spanning the full range of severity levels on each DLQI item (see later). Participants in the valuation task were faced with five of these health states, each described in words according to its array of DLQI severity levels. Health states were presented in randomized order, and participants had to valuate them successively, one at a time.

### Time trade-off valuation

The outcome measure concerning the valuation task was the TTO utility on each health state presented. The TTO valuation method establishes subjective utility values for impaired health conditions by asking respondents to hypothetically trade off their length of life for their quality of life [2].

We used a 10-year time frame, which is a widely adopted method in valuation studies [21]. Individuals were asked to imagine having a remaining lifespan of ten years, which they were to live in a given hypothetical health state. Then they had to indicate how many of these ten years they would be willing to give up in exchange for regaining perfect health for the rest of their lives. There were 21 response categories ranging from 0 to 10 years by half-year increments. The procedure did not include a ‘worse than dead’ task, i.e. relinquishing one’s entire remaining life was the lowest valuation available. As regards preference elicitation, participants were asked to indicate their point of indifference by moving a horizontal slider from its initial value of 5 years to the left or right (i.e. towards lower or higher values) in half-year increments. The position of the slider was reset to its midpoint (5 years) before the valuation of each health state (“Appendix A.2”).

As a last step of the valuation procedure, [0–1] utility values [*y*] were calculated for each response according to the formula

1 $$y = {1} - t/{1}0$$

whereby [*t*] was the respondent’s choice in the TTO valuation task, i.e. the number of years he/she would be willing to trade off for perfect health.

### Study design

Two important aspects of the study design were: selecting the health states for the valuation task, and assigning sets of randomly chosen health states to participants.

#### Selection of health states

The full set of health states was compiled as the union of two subsets (Additional file 1: Table S19). The first subset, consisting of 64 states, was selected following an orthogonal design, in a way to satisfy the following two criteria: (1) for all ten DLQI items the full range of severity levels were uniformly represented across health states; (2) the severity scores on all ten DLQI items were pairwise uncorrelated. This core subset included a health state with minimal HRQoL impact (H23; DLQI score = 1)^1^ as well as a ‘worst possible’ health state (H73) bearing a maximal negative impact on all areas of life (DLQI score = 30). The other 62 health states had a DLQI total score between 10 and 20, with a mean of 15.00 and a standard deviation of 2.38.

The second subset consisted of 9 health states, three of which (H70–72) were taken from a similarly designed previous study [9]. The other six health states (H01; H65–69), all representing milder skin conditions (DLQI scores between 1 and 5) were selected as the six most frequent actual health states reported by a joint sample of 838 patients surveyed in four cross-sectional studies carried out by our research team [22–25].

#### Block design and randomization

Participants were randomly assigned to one of 18 experimental conditions (‘random blocks’) determining the five health states to be valuated. The random assignment method was meant to ensure that health state characteristics were independent of subject characteristics. Health states within each block were presented in random order.

As for the composition of random blocks, the ‘worst possible’ state (H73) was included in all 18 blocks, whereas the four other health states were selected randomly from four predefined clusters of health states, more-or-less homogeneous in terms of their DLQI total scores (Additional file 1: Table S10). This was meant to ensure that the set of health states in each random block spanned a comparable range of severity levels. However, this objective wasn’t entirely met due to substantial variability concerning the severity of the mildest (#1) state in each random block, with DLQI scores varying between 1 and 12.

### Sample exclusions

Preliminary analyses indicated that the initial data set was of insufficient quality for defining a societal value set. Apparently a large proportion of participants didn’t take the time to complete the valuation task to any reasonable standard. This was evident from the following observations. (1) Response times per health state were 5 s or less in 21% and 10 s or less in 39% of valuation instances. (2) The within-subject standard deviation of [0–1] utilities across the five health states was zero for 31% and less than 0.1 for 63% of respondents. (3) Many respondents gave inconsistent valuations, i.e. they assigned lower utilities to some of the milder or medium severity health states than to the ‘worst possible’ state. Thus, it was necessary to restrict the sample and define inclusion criteria concerning the main statistical analyses. Respondents were screened on response times as well as on the consistency and informativeness of their valuations (Table 1).

**Table 1:** Overview of sample restrictions

Survey completed by	2459
Excluded due to quota requirements	− 458
Initial full sample	2001
Non-traders (handled separately)	− 317
Initial sample (traders only)	1684
Excluded due to identical valuation on all five health states	− 296
Excluded by combination of response time and response consistency criteria	− 656
Excluded due to uninformative responses	− 207
Final sample used for the regression analysis (traders only)	525
Non-traders (used for the adjustment of regression results)	+ 317
Respondents whose valuations were taken into account in defining the value set	842

#### Exclusion of subjects with all identical responses

We excluded from the sample 296 individuals who gave the same valuation on all five health states because their responses had no information value concerning our main research objective. However, we handled separately those 317 ‘non-trader’ individuals who gave a valuation of 1 on all five health states, i.e. those who were not willing to trade off any of their lifespan for being cured of even the most severe of skin diseases. Whereas it would have been pointless to include non-traders’ data in the main statistical analyses, it was reasonable and well justified to take their valuations into account in defining a societal value set.

#### Exclusion by response time

Exclusion due to too quick responses was considered in relation to the shortest (‘min’) and the median (‘med’) response time concerning the five valuations made by an individual.^2^ Lacking of an a priori criterion, we experimented with different combinations of exclusion thresholds: [thr_min] varying in the range [4–12 s] and [thr_med] varying in the range [8–24 s]. We performed two nested classification analyses to select these two exclusion thresholds conjointly with a third threshold concerning the maximum tolerable inconsistency of responses (Additional file 1: S.1). We settled with [thr_min = 5] and [thr_med = 10], implying the exclusion of participants whose shortest response time was 5 s or less and whose median response time was 10 s or less.

#### Exclusion by response inconsistency

As a minimal requirement of response consistency we expected that participants should assign the lowest utility to the ‘worst possible’ state (H73) and all other health states should be assigned higher or equal values. However, this expectation was violated by nearly half of those respondents whose valuations exhibited any variability at all across the five health states. So we concluded that requiring a non-negative utility difference with respect to the ‘worst possible’ state would be too strict a criterion.

Thus, we experimented with softer criteria, requiring that all utility differences with respect to state H73 should exceed a certain threshold [thr_diff], which we varied in the range [− 0.40 to 0.00]. Again, this threshold (conjointly with the response time thresholds) was determined as the outcome of two nested classification analyses (Additional file 1: S.1). We settled with [thr_diff = (− 0.10)], implying the exclusion of participants whose valuations on any of the milder/moderately severe health states was more than 0.10 lower than their valuations on the ‘worst possible’ state.

#### Exclusion due to uninformative responses

The thus far reduced sample still contained respondents whose valuations exhibited low within-subject variability without having any meaningful information content. Hence we introduced an additional screening criterion to filter out individuals whose valuations were both partially inconsistent and of minimal variability. This was operationalized as follows: a set of valuations was considered lacking of any meaningful information if the respondent only used two different values in his/her valuations and he/she assigned the higher of these to the ‘worst possible’ state.

Applying this criterion resulted in the exclusion of further 207 participants, so that our final sample consisted of *n*_TR_ = 525 trader and *n*_NT_ = 317 non-trader individuals. Interestingly, this complementary criterion eliminated all individuals whose valuations on the five health states were to any degree inconsistent, i.e. it had the same effect as choosing a value of [thr_diff = 0] for the minimally required utility difference with respect to the ‘worst possible’ state.

### Regression analysis

We performed regression analyses to explore how the TTO valuation of health states was related to [0–3] severity levels concerning the ten items of the DLQI. On each item the zero severity level (no impact on quality of life) was considered the baseline, and levels 1, 2, 3 were represented by three separate dummy variables. Thus, the full set of regressors consisted of 10 × 3 = 30 dummy variables.

We used incremental dummy coding so that the regression coefficient on the dummy for a particular severity level represented the incremental disutility with respect to the previous (one lower) level. As for the estimation method, random effect estimation was applied throughout the analysis, as it was consistent with the randomized block design, and as its applicability was confirmed by the Hausman-test.

#### Initial model types

We used four initial types of regression models: (1) linear model; (2) censored linear model; (3) ordinal regression; (4) beta regression. In addition, given the large individual differences in respondents’ valuation scale usage, we developed three versions of a two-part scalable model which were suitable for accommodating this form of heterogeneity: (5) scalable linear model; (6) scalable censored model; (7) scalable beta regression.

The linear model, serving as a point of departure, was judged unsatisfactory because of its assumption concerning a continuous and unconstrained range of values for the dependent variable. This assumption was violated in our research as response options in the TTO valuation task were confined to the set {0; 0.5; …; 9.5; 10}, and the corresponding utility values were constrained to the interval [0–1]. For this reason we also considered censored, ordinal, and fractional dependent variable models, which are more suitable for normalized utility valuations than the linear model.

As regards censored regression, we applied two-sided censoring of the dependent variable [*y*] with [*y*_L_ = 0] as the lower bound and [*y*_U_ = 1] as the upper bound. This might appear paradoxical at first because assigning a utility greater than 1 to a health state is intrinsically meaningless. In practice, however, due to idiosyncratic perturbations inherent to respondents’ behavior, observing valuations greater than 1 would have been probable had the rating scale been open-ended. Indeed, estimating a censored regression model with normally distributed errors revealed that in 13.0% of cases right-censoring was effective, i.e. in the absence of an upper bound the person would have assigned a utility greater than 1.

We also estimated ordinal (probit) regression models as another way to accommodate the fact that idiosyncratic perturbations could only have a limited effect on TTO valuations due to the constrained set of response categories. Ordinal models imply a mapping between a continuous-valued latent variable [y^*^] and the observed outcomes [*y*], whereby a right-unbounded upper interval is mapped to the highest and a left-unbounded lower interval is mapped to the lowest outcome category, with a number of intervals in between. We found that the thresholds between the underlying latent variable intervals were close to uniformly spaced, therefore the use of equidistant ordinal models was appropriate.

We also applied beta regression as a third approach to modeling [0–1] constrained TTO valuations. Such models, which specify a beta type conditional distribution concerning a fractional dependent variable, have previously been used to model the relationship of HRQoL outcomes to health condition characteristics, treatment options, and socio-demographic or other individual-specific features [26, 27]. Following the usual parametrization of beta regression models, two sets of regression coefficients and two link functions are required to describe the effects of the regressors on (1) the conditional mean of the distribution and (2) a precision parameter, which is inversely related to the conditional variance of the distribution. After experimentation,^3^ we opted for the basic model version imposing a constant precision parameter, and we settled with the probit link specification concerning the conditional mean.

#### Two-part scalable models

The idea of developing a scalable model originated from the observation that the individual-specific error component in the random intercept linear model exhibited a substantially negatively skewed distribution (skewness = − 0.72). This suggested an asymmetric tendency in respondents’ behavior, with most respondents’ valuations being confined to a relatively narrow upper region and only a minority of individuals using the lower regions of the utility scale.

To incorporate this heterogeneity to the model, we separated from the between-subject variability of effectively used scale ranges the relative position of each individual’s valuations within his/her effective scale range, which was further analyzed in relation to health state characteristics. As a result, the following two-part scalable model was constructed:

2 $$y = {1} - \lambda z$$

3 $$z = \alpha + x^{\prime}\beta + u + v$$

whereby [*y*] is the TTO utility assigned to some health state, [*λ*] is the effective scale range used by the individual, [*z*] is the relative disutility from the health state, expressed in proportion to the effective scale range, [*x*] is the regressor vector representing the health state characteristics, [*β*] is the corresponding regression coefficient vector, [α] is the global intercept, [*u*] is the individual-specific random intercept, and [*v*] is the idiosyncratic error term.

The effective scale range was conceptually an unobserved individual factor of heterogeneity in the model. Yet, by imposing the natural assumption *z*(H73) = 1, a proxy was obtained in the form

4 $$\lambda^{*} = {1} - y\left( {{\text{H73}}} \right)$$

which was directly observable. Then, after performing the transformation

5 $$z^{*} = \left( {{1} - y} \right)/\left( {{1} - y\left( {{\text{H73}}} \right)} \right)$$

for all health states valuated by an individual, the linear model (Eq. 3) could be estimated on the pooled set of [$z^{*}$] values.^4^

Estimated mean utilities (conditionally on health state characteristics) were obtained by combining the two model components, i.e. the regression relationship (Eq. 3) and the distribution of [*λ*] across individuals. As implied by model types (5), (6), and (7), Eq. (3) was estimated using ordinary linear, beta, and censored linear regression. As to the latter, we only used left-censoring (i.e. censoring at *z*_L_ = 0) because after the relative disutility *z*(H73) had been normalized to 1 for each individual, *z*_U_ = 1 was not active as a right-censoring threshold.

#### Model selection

As regards the seven types of models presented earlier, the simple and scalable linear models were judged insufficient for accurately capturing the relationship between DLQI scores and TTO valuations. Nonetheless, for the sake of completeness these models, too, were estimated and evaluated. Choice between the ordinal, censored, beta, scalable censored, and scalable beta regression models was made on the basis of model performance indicators. As of the latter, we used linear correlation coefficients and mean absolute deviations, both of which were calculated with respect to individual valuations, mean utilities, and median utilities.

To select the optimal set of regressors, as a starting point we specified that all non-zero severity levels of each DLQI item must have negative or zero incremental effects on the predicted TTO utility. Variable selection was carried out in two steps. First, starting from the initial model we did backward elimination until we arrived at a maximal model version consistent with the theoretical prerequisites, i.e. a model with the largest set of variables all having negative coefficients. In the second step we used cross-validation methods to increase the robustness and generalizability of the model by removing further variables.

#### Model cross-validation

We performed cross-validation analyses concerning all different model types and model versions. Following the procedure by Rand-Hendriksen et al. [28], all regression models were estimated on 18 different subsamples, each containing the data of individuals in 17 of 18 random blocks. The model estimated on each subsample was used to extrapolate the valuations made by individuals in the left-out random block. Finally, the extrapolated values were pooled across subsamples and compared with the observed valuations. In addition to calculating cross-validation fit indices, we examined the range of estimated coefficients on each variable and reported minimum and maximum values across the 18 subsamples (Additional file 1: Tables S17, S18). This allowed us to impose stricter, cross-validated non-positivity criteria.

#### Value set construction

We constructed an experimental value set providing predicted TTO utilities for any combination of DLQI severity levels. This was carried out in two steps: (1) calculating predicted utilities for ‘trader’ individuals, and (2) adjusting for non-traders’ valuations.

The first step involved mapping combinations of DLQI severity levels to traders’ utilities according to the vector of estimated regression coefficients. This was carried out in different ways depending on the type of regression model (Additional file 1: S.2).

Predicted utilities concerning the total general population [${\hat{y}}_{a}$] were calculated in the form of a weighted average between traders’ and non-traders’ valuations:

6 $${\hat{y}}_{a} = w \cdot 1 + (1 - w) \cdot {\hat{y}}$$

whereby [${\hat{y}}$] denotes traders’ predicted valuation, non-traders’ valuation is 1 for any health state, and [*w*] is the proportion of non-traders.

## Results

### Subject characteristics

The composition of both the original (*n* = 2001) and the reduced sample (*n*_R_ = 842) was broadly matching that of the adult general population in terms of gender, age, place of residence, geographic region, and employment status (Additional file 1: Tables S2–S6). As regards marital status and education, the sample exhibited more substantial deviations from the population (Additional file 1: Tables S7, S8); in particular, individuals in the lowest category of education (primary school or less) were strongly underrepresented (initial sample: 5.7%, reduced sample: 4.4%, population: 28.0%).

#### Effects of sample exclusions

The screening procedure was successful in enhancing the quality of the sample in terms of response times and consistency of valuations (Additional file 1: S.3), whereas the composition of the sample was altered to a lesser extent (Additional file 1: Tables S2–S8). Analyses conducted in the latter regard gave evidence for very weak associations, with the Cramer coefficient (*C*) taking on values less than 0.10 and the chi-square test of independence indicating in most cases a non-significant relationship (*C* = 0.005; *χ*^2^(1) = 0.05; *p* = 0.817 for gender, *C* = 0.031; *χ*^2^(3) = 1.87; *p* = 0.601 for place of residence, *C* = 0.032; *χ*^2^(2) = 2.06; *p* = 0.357 for geographic region, *C* = 0.075; *χ*^2^(4) = 11.39; *p* = 0.023 for marital status, *C* = 0.090; *χ*^2^(8) = 16.33; *p* = 0.038 for employment status).

Nonetheless, sample exclusions had statistically significant effects on age (*C* = 0.098; *χ*^2^(5) = 19.04; *p* = 0.002) and education (*C* = 0.077; *χ*^2^(2) = 11.96; *p* = 0.003). In particular, the proportion of middle aged and older individuals (age > 45 years) increased from 56.5% to 60.8%, and the proportion of individuals with college or university degree education increased from 18.9% to 22.0%.

#### Variations in the proportion of non-traders

We examined whether the three categories of participants in the valuation task (traders, non-traders, and those excluded from the sample) were evenly distributed across the 18 random blocks. The proportion of excluded subjects varied between 50.9% and 69.1% (coefficient of variation: CV = 9.3%), and there was no significant heterogeneity across random blocks (*χ*^2^(17) = 23.87; *p* = 0.123). In contrast, we found substantial heterogeneity concerning participants’ non-trader behavior. The proportion of non-traders varied between 7.8% and 26.9% (CV = 32.4%), and the null-hypothesis of homogeneity was rejected (*χ*^2^(17) = 39.50; *p* = 0.0015).

We explored possible sources of this heterogeneity and found as a likely explanation non-trivial differences in the composition of random blocks, which was manifested in differing ranges of DLQI scores over the set of five health states presented per block. The reason for this was the substantial inequality concerning the severity of the mildest health state in each random block, with DLQI scores on state #1 ranging between 1 and 12 (Additional file 1: Table S10). Indeed, the proportion of non-trader subjects was positively correlated with the DLQI score on health state #1 (lin. corr. = 0.415; *p* = 0.087), implying that individuals faced with a less diverse set of health states were less likely to engage in the time trade-off.

### Results of the valuation task

Concerning the restricted sample of ‘trader’ respondents (*n*_TR_ = 525), utility valuations varied substantially across health states (between-groups st. dev. = 0.100) as well as across individuals (within-groups st. dev. = 0.241). The association between health states and valuations was relatively weak (*η*^2^ = 0.148), nonetheless statistically significant (Kruskal–Wallis *χ*^2^(72) = 399.17; *p* = 7.8E-47).

#### Valuation of health states

**Fig. 1:**
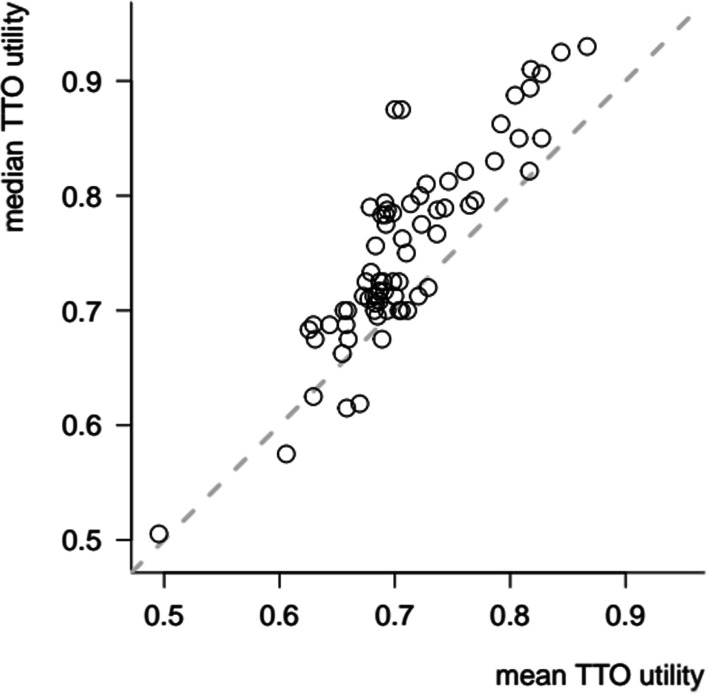
Mean and median utility valuations on the 73 health states involved in the study

Mean TTO utilities varied between 0.496 (H73: ‘worst possible’ state) and 0.867 (H65: state with minimal HRQoL impact); (Additional file 1: Table S19). Median utilities varied between 0.505 and 0.930, and were strongly positively correlated with mean utilities (lin. corr. = 0.875; *p* = 4.2E−24). Medians were (with a few exceptions) systematically higher than the means, indicating a negatively skewed distribution across individuals (Fig. 1). This was especially the case for the milder health states, which were assigned the highest possible utility (*y* = 1) by a substantial proportion of respondents.

As for the relationship between severity levels and TTO valuations, mean utilities were strongly negatively correlated with the DLQI total score of health states (lin. corr. = − 0.792; *p* = 7.1E−17), which by itself accounted for 62.7% of the total variance. Nonetheless, 37.3% of the variance was left unexplained, so there was scope for improving the fit by taking into account the severity levels on each DLQI item. Individual TTO valuations were to a moderate extent (yet significantly) negatively correlated with DLQI total scores (lin. corr. = − 0.359; *p* = 0.0012), resulting in *R*^2^ = 0.129, i.e. 12.9% of total variance explained.

#### Effective scale range

**Fig. 2:**
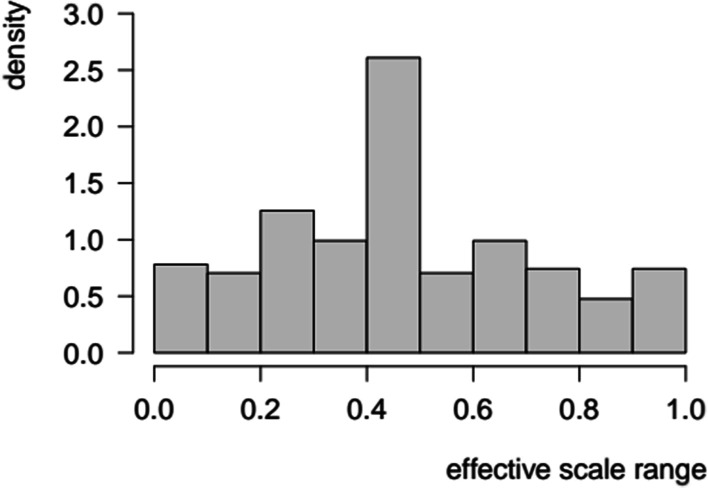
Frequency distribution of effective scale ranges concerning ‘trader’ subjects

The range of values spanned by individuals’ valuations exhibited substantial variability. Concerning the restricted sample (*n*_TR_ = 525), the effective scale range varied between 0.05 and 1.00, with a mean of 0.504, a median of 0.495, and a standard deviation of 0.261. The distribution was roughly symmetrical around the modal value of 0.500 (Fig. 2).

### Regression results

**Fig. 3:**
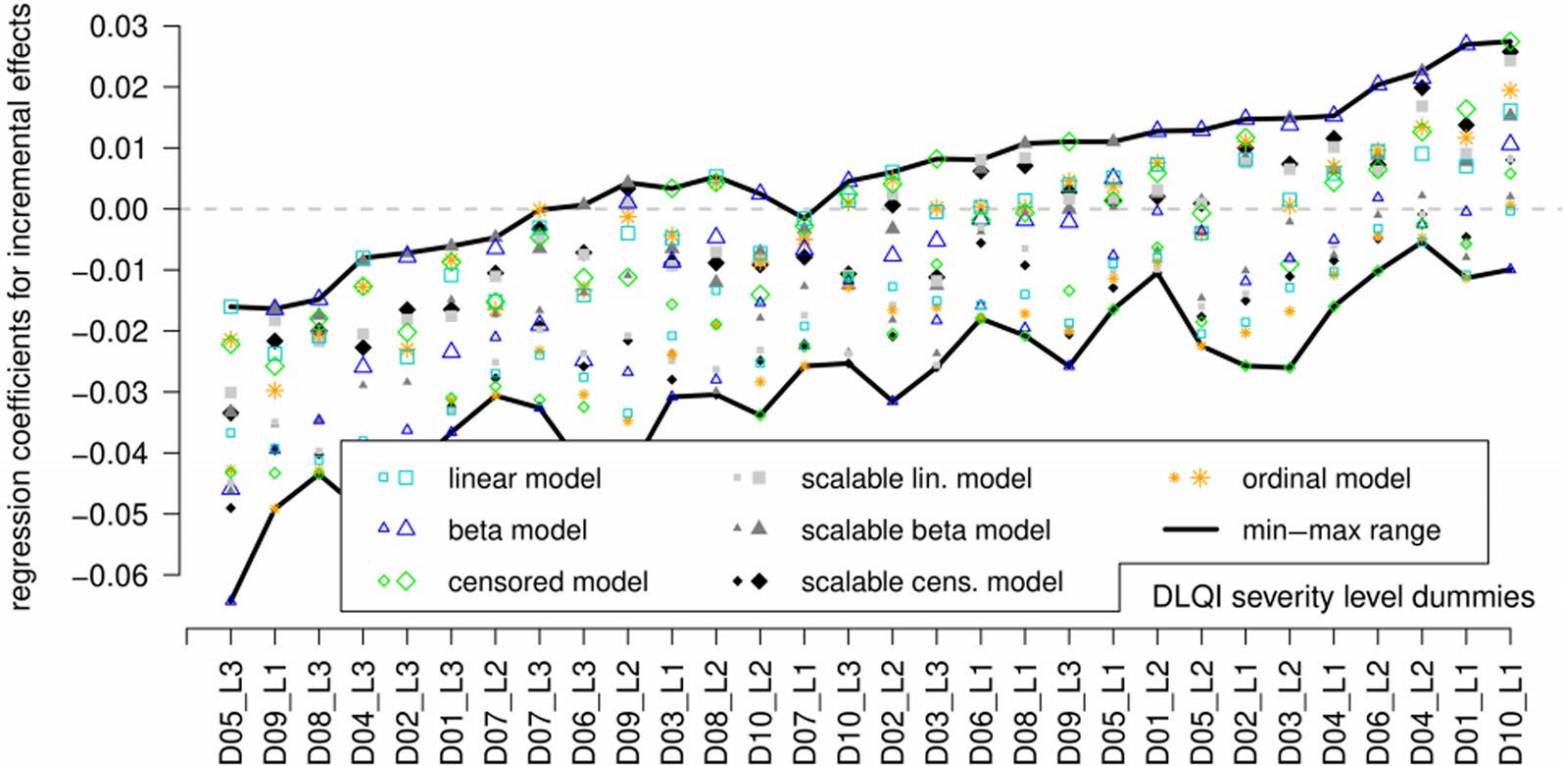
Min–max range of regression coefficients over 18 cross-validation subsamples concerning the initial versions of the seven regression models

The final set of explanatory variables was obtained in two steps. First, starting from the initial model (which was estimated and cross-validated for all seven model types; Fig. 3), seven dummy variables were omitted, all of which had positive but statistically non-significant coefficients. Thus, an intermediate model version was obtained, which was consistent with our theoretical prerequisites, yet not optimal in terms of robustness.

**Fig. 4:**
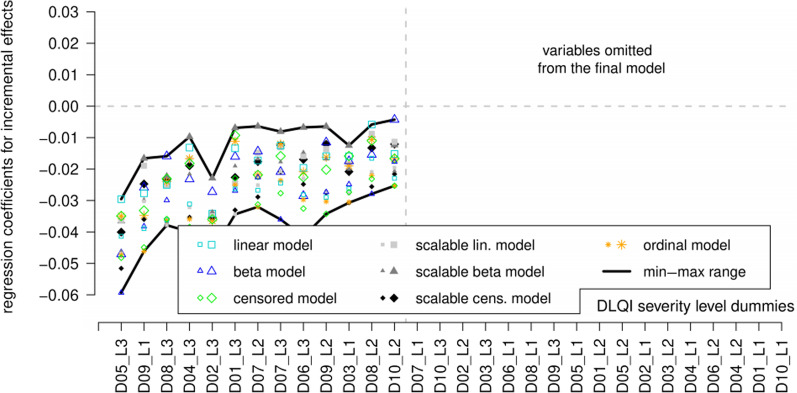
Min–max range of regression coefficients over 18 cross-validation subsamples concerning the final versions of the seven regression models

In the second step altogether 24 model versions were considered and compared on multiple cross-validation criteria (Additional file 1: Table S11). The final model was obtained through the omission of ten further variables whose coefficients, although overall negative, took on positive values in some of the cross-validation subsamples. The final model only contained variables whose coefficients were negative throughout all cross-validation subsamples in all seven types of models (Fig. 4).

#### Cross-validation outcomes

Cross-validation fit indices improved substantially along the model selection procedure (Additional file 1: S.4; Additional file 1: Tables S12–S14). Reducing the set of predictor variables was also instrumental for dealing with the issue of model overfitting. Comparing the cross-validation fit indices with the ‘full sample’ fit indices revealed that both the initial and the intermediate model versions largely overfitted the sample, whereas the degree of overfitting was much lower for the final model.

#### Performance indicators

Concerning the final model versions, linear correlation coefficients between the fitted TTO utilities and the observed mean values were between 0.835 and 0.862, depending on the type of model (Table 2). This corresponds to *R*^2^ values of 0.697 to 0.743, i.e. up to 74.3% of the variability in mean valuations was explained by the model with 13 variables, a substantial improvement with respect to *R*^2^ = 0.627 concerning the uni-dimensional model which uses DLQI total score as a single predictor variable.

**Table 2:** Measures of full sample fit for the seven types of regression models (final versions)

	Linear	Beta	Censored	Ordinal	Scalable linear	Scalable beta	Scalable censored
Linear correlation coefficient w.r.t							
Individual TTO valuations	0.369	0.367	0.369	0.369	0.369	0.365	0.369
Mean TTO utilities	0.862	0.835	0.857	0.856	0.859	0.837	0.855
Median TTO utilities	0.786	0.778	0.787	0.784	0.776	0.763	0.778
Mean absolute difference w.r.t							
Individual TTO valuations	0.198	0.198	0.197	0.197	0.197	0.200	0.196
Mean TTO utilities	0.024	0.031	0.029	0.029	0.026	0.034	0.031
Median TTO utilities	0.056	0.049	0.049	0.049	0.052	0.048	0.047

Fit to individual valuations was much weaker, as was indicated by linear correlation coefficients of 0.365–0.369 and corresponding *R*^2^ values up to 0.136. Nonetheless, given the low degree of association between individual valuations and health states (*η*^2^ = 0.148) to start with, in relative terms our model achieved a performance of 0.136/0.148 = 91.8%. Also, our model accounted for 36.9% of the incremental explanatory power of a maximal benchmark model (representing each health state by a separate variable; *R*^2^ = 0.148) over the uni-dimensional model (containing DLQI total score as the single predictor; *R*^2^ = 0.129).

The model also performed well in terms of the difference between fitted and observed mean utilities. The mean absolute difference (MAD) ranged between 0.024 and 0.034, depending on model type. Differences with respect to individual valuations were much larger, in the order of 0.200.

#### Comparison and choice between model types

**Fig. 5:**
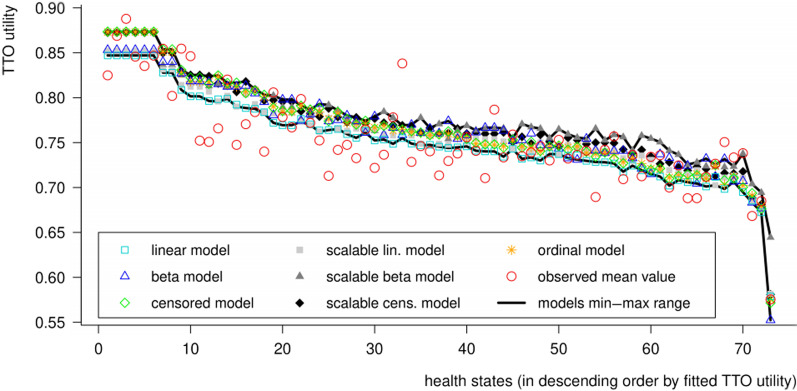
Fitted and observed mean TTO utilities across the 73 health states involved in the study

Comparing the ‘full sample’ MAD values across the seven types of models revealed three salient tendencies: (1) linear models were the best fitting to the observed means, whereas ordinal and censored models were the best fitting to the observed medians; (2) whether linear, censored, or beta regression models being concerned, using a scalable variant improved the fit to the medians and worsened the fit to the means; (3) beta regression models achieved relatively poor fit to mean valuations and were altogether outperformed by censored models. However, these differences were relatively small, and the utilities fitted by different types of models varied closely together across health states (Fig. 5).

As regards the type of regression model to be used for determining the TTO value set, we opted for the censored models, which we considered optimal for two reasons. First, censoring at the maximal utility (*y*_U_ = 1) appeared necessary, as was indicated by the asymmetric distribution of valuations on the relatively mild health states. Second, within the seven types of models considered, the censored models provided the best fit to the median TTO values, which we judged as important as the fit to the means. As for the choice between the simple and the scalable variants, we decided to calculate average coefficients across the two model variants.

### Utility impact of DLQI items

The final model versions contained 13 dummy variables, all with negative regression coefficients (Table 3; p-values are reported in Additional file 1: Table S15). This means that each DLQI item was found to exert a significant negative effect on the valuation of health states, although partial effects were not perfectly distinguishable across the three severity levels.

**Table 3:** Output for the seven types of regression models^a^ (final versions^b^)

	Linear		Beta		Censored		Ordinal		Scalable linear		Scalable beta		Scalable censored	
	coeff^c^	SE	coeff^d^	SE	coeff^c^	SE	coeff^c^	SE	coeff^c^	SE	coeff^d^	SE	coeff^c^	SE
Intercept	81.82	1.15	82.56	NA	84.96	1.24	84.93	NA	82.39	0.85	82.29	NA	84.82	0.88
D01_L3	− 1.96	0.95	− 2.04	1.99	− 1.69	1.14	− 1.83	1.06	− 2.84	0.86	− 1.23	0.99	− 2.73	0.91
D02_L3	− 3.83	0.74	− 3.55	1.40	− 4.09	0.91	− 4.12	0.86	− 3.81	0.76	− 2.74	0.77	− 3.97	0.79
D03_L1	− 2.27	0.71	− 2.06	1.19	− 2.34	0.87	− 2.64	0.82	− 2.42	0.75	− 1.66	0.69	− 2.68	0.76
D04_L3	− 2.55	0.75	− 3.51	1.47	− 3.21	0.94	− 3.00	0.88	− 2.78	0.76	− 1.71	0.74	− 3.07	0.81
D05_L3	− 3.39	0.75	− 5.29	1.40	− 3.98	0.93	− 3.96	0.88	− 4.12	0.77	− 4.16	0.77	− 4.57	0.81
D06_L3	− 2.47	0.74	− 3.74	1.38	− 2.82	0.91	− 2.64	0.87	− 2.03	0.75	− 1.18	0.72	− 2.18	0.81
D07_L2	− 2.29	0.82	− 1.87	1.20	− 2.72	0.96	− 2.75	0.92	− 2.06	0.83	− 1.25	0.82	− 2.40	0.85
D07_L3	− 1.88	0.99	− 2.47	1.81	− 2.17	1.17	− 1.83	1.11	− 1.71	0.97	− 1.28	0.98	− 1.64	1.02
D08_L2	− 1.18	0.82	− 2.06	1.42	− 1.72	1.01	− 1.64	0.97	− 1.42	0.86	− 1.39	0.86	− 1.84	0.90
D08_L3	− 3.10	0.98	− 2.38	1.71	− 3.01	1.18	− 3.08	1.13	− 3.15	1.00	− 3.09	1.02	− 3.07	1.04
D09_L1	− 3.14	0.88	− 3.12	1.41	− 3.73	1.05	− 3.91	1.01	− 2.27	0.91	− 2.25	0.86	− 2.90	0.94
D09_L2	− 1.96	0.85	− 1.84	1.42	− 2.36	1.01	− 1.98	0.97	− 1.89	0.86	− 1.15	0.84	− 1.85	0.89
D10_L2	− 1.85	0.67	− 0.91	1.15	− 2.03	0.80	− 2.01	0.77	− 1.58	0.69	− 1.69	0.69	− 1.70	0.71

^a^Figures in the table are in units of 0.01

^b^Chosen model types: censored, scalable censored

^c^Coefficients represent the incremental disutility with respect to the previous level of the DLQI item

^d^Beta regression coefficients are rescaled in order to represent average partial effects

**Fig. 6:**
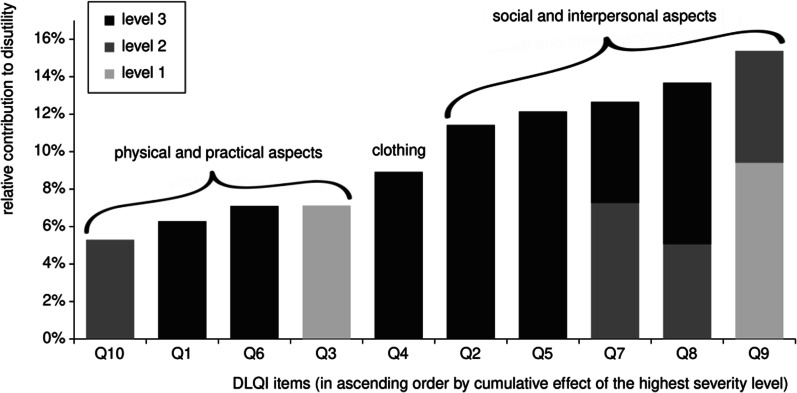
Relative contributions of DLQI items to the total disutility from the ‘worst possible’ health state

As regards the overall negative relationship between DLQI scores and TTO utilities, the regression results indicated substantial differences across DLQI items as well as across severity levels (Fig. 6). The relative contribution of DLQI items (in proportion to the total disutility from the ‘worst possible’ health state) varied between 5.3% and 15.4%. Furthermore, the relative contribution of distinctive severity levels within the cumulative effect of each item varied between 0 and 100%.

Examining the cumulative effects (Table 4) revealed that the largest negative impacts on TTO utility (− 0.034 to − 0.046) were all related to the social and interpersonal consequences of skin diseases: embarrassment and self-consciousness [Q2], social and leisure activities [Q5], work and school [Q7], close personal relationships [Q8], and sexuality [Q9]. In contrast, the smaller effects (− 0.016 to − 0.021) were all related to the physical and practical aspects: pain and itching [Q1], shopping and house chores [Q3], sports [Q6], and problems caused by treatment [Q10]. Clothing [Q4], the only DLQI item with both a practical and a social aspect, had an effect size in between (− 0.026).

**Table 4:** Cumulative partial effects for calculating predicted TTO utilities for DLQI health states

DLQI item	Partial effects^a^ for traders			Partial effects^a^ adjusted for non-traders		
	Level 1	Level 2	Level 3	Level 1	Level 2	Level 3
Q1	0.000	0.000	− 0.022	0.000	0.000	− 0.019
Q2	0.000	0.000	− 0.040	0.000	0.000	− 0.034
Q3	− 0.025	− 0.025	− 0.025	− 0.021	− 0.021	− 0.021
Q4	0.000	0.000	− 0.031	0.000	0.000	− 0.026
Q5	0.000	0.000	− 0.043	0.000	0.000	− 0.036
Q6	0.000	0.000	− 0.025	0.000	0.000	− 0.021
Q7	0.000	− 0.026	− 0.045	0.000	− 0.022	− 0.038
Q8	0.000	− 0.018	− 0.048	0.000	− 0.015	− 0.041
Q9	− 0.033	− 0.054	− 0.054	− 0.028	− 0.046	− 0.046
Q10	0.000	− 0.019	− 0.019	0.000	− 0.016	− 0.016
Intercept	0.849			0.873		

^a^Partial effects represent the cumulative disutility from distinctive levels of DLQI items

### Experimental value set

Defining a value set for the DLQI was straightforward once estimates for the regression intercept and the partial effects of the regressors were available. Starting from the model parameters estimated for traders, parameters adjusted for non-traders’ valuations were calculated according to Eq. 6 (Table 4). Then, the predicted TTO utility for any specific health state was easy to obtain by summing the adjusted partial effects of the ten DLQI items, each according to its level of severity, and adding this summed negative value to the intercept (“Appendix A.3”).

**Fig. 7:**
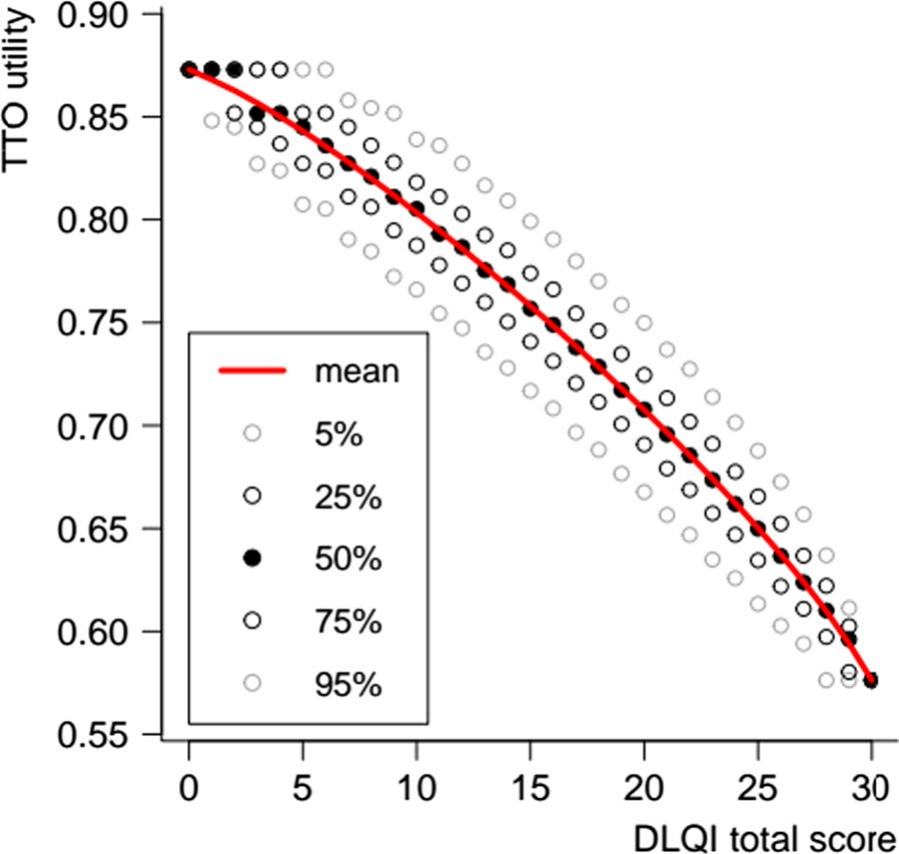
Conditional distribution of predicted TTO utilities across all possible combinations of DLQI severity levels

As an illustration, we calculated the predicted utility for all possible combinations of severity levels and plotted its conditional percentiles against the DLQI total score (Fig. 7). Even though the mathematically possibly 4^10^ combinations aren’t representative of the empirical distribution of severity levels across real-life dermatological conditions, our calculations suggest that the overall relationship between DLQI scores and TTO utilities is concave rather than linear.

**Fig. 8:**
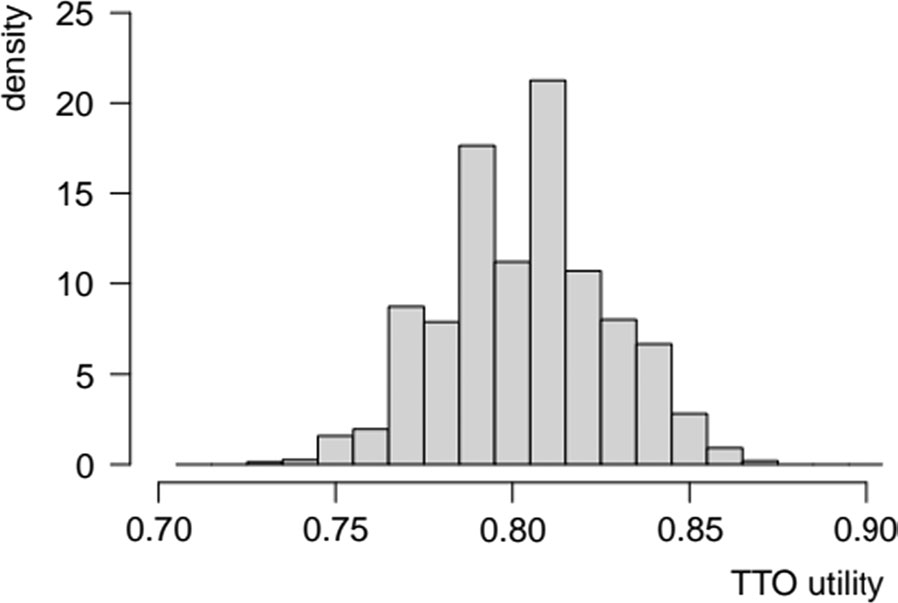
Distribution of predicted TTO utilities across health states with DLQI total score = 10

In addition, we plotted the distribution of predicted utilities across health states with a DLQI total score of 10 (Fig. 8), which is the habitual threshold for access to publicly financed dermatological treatments in many healthcare systems. These analyses gave evidence of substantial variability in TTO utilities across health states with a given DLQI score, e.g. for DLQI = 10 the predicted utilities exhibited an interquartile range of 0.031 and a difference of 0.073 between the 5th and the 95th percentiles.

## Discussion

This study is the first attempt to develop a utility value set for health states evaluated on the DLQI scale. Our results have both methodological and health economic bearing, the former being concerned with issues of research design and statistical modeling, and the latter having implications for health care policy.

### Screening of participants

Due to the unsatisfactory initial data quality, it was indispensable to screen participants based on their behavior in the valuation task. This resulted in a significantly better quality final sample, albeit at the cost of discarding the data from 69% of ‘trader’ subjects. As to this latter point, the exclusion of individuals may compromise the representativeness of the sample [29, 30], which could have caused serious problems in constructing a value set. Fortunately the screening procedure didn’t cause substantial changes in the socio-demographic composition of the sample.

### Handling of non-traders

The separate handling of non-trader respondents emerged as a compromise between two conflicting considerations. On the one hand, assigning a utility of 1 to all health states could be attributed to valid ethical, religious, or spiritual considerations, so it was justified that non-traders’ valuation should be represented appropriately in the societal value set. On the other hand, non-traders’ responses didn’t contain any information as regards how the variation in individuals’ valuations was related to health state characteristics, so it was better not to use their data in the regression analysis.

The separate handling of non-traders offered several advantages. First, adjusting for non-traders’ valuations in the model parameters and the resulting value set was straightforward by means of a linear transformation. Second, non-traders’ exclusion from the main statistical analyses eliminated possible biases which could have arisen due to their uneven distribution across experimental conditions. Third, in case the estimated proportion of non-traders proved to be imprecise, ulterior corrections could easily be made once a better estimate became available.

As a related issue, further screening of non-traders would have been useful. The negative relation which we found between the variability of DLQI total scores and the proportion of non-traders in different random blocks suggests that participants’ non-trader behavior wasn’t completely exogenous but rather it was influenced by the health states presented in the valuation task. In particular, some of the ‘non-traders’ may have chosen to give all the highest values not for ethical or spiritual reasons but for lack of interest about or difficulty in assessing hypothetical health states, and it would have been better to exclude these respondents from the sample altogether. This would have required the use of additional screening questions concerning their motives for not engaging in the ‘quality for time’ trade-off.

### Regression modeling

We departed in important respects from the classical linear regression model. In addition to using censored, ordinal, and beta regression, we worked with scalable, two-part models of our own design.

We argued for the necessity of using censored or ordinal models on the ground that the utility scale was inherently bounded at the top, which had considerable effects on the conditional distribution of TTO valuations. We found that the partial effects of DLQI items were underestimated in the linear model, as it was essentially fitted to the mean valuations, which exhibited smaller variability in comparison with the medians. In contrast, censored and ordinal models were able to extract more information from the capped valuations, which resulted in larger partial effects. We expected that beta regression should offer an equally efficient solution to the same problem but found it produced inferior model fit statistics in comparison with either the censored or the ordinal model.

Our two-part scalable models offered important benefits. First, the relationship between the DLQI characteristics and the relative disutilities of health states was more accurately estimable than the original relationship concerning the observed TTO utilities. As a second benefit, scalable models offer the possibility of ulterior readjustment in case a better estimate for the population distribution of effective scale ranges becomes available. In such a case, the predicted TTO utilities can easily be adjusted by re-weighting the relative disutilities according to the updated distribution.

### Impact on health-related quality of life

The results shed light on important aspects of how individuals’ HRQoL was affected by the negative consequences of dermatological conditions.

#### Factors of dermatological disease burden

We examined how utility valuations were affected by different types of discomforts caused by skin diseases. We found a definitive structure consisting of two clusters, with DLQI items belonging to either social/interpersonal or physical/practical aspects of HRQoL, and ‘clothing’ constituting a unique category in between. Cumulative disutilities associated with the highest severity level of each DLQI item were more-or-less homogeneous within each cluster and markedly disparate between the two clusters, social/interpersonal aspects being roughly twice as important as the physical/practical aspects.

#### Increasing marginal disutility

We obtained tentative results about how the utility impact of dermatological health states was related to their overall severity. Aggregating the predicted TTO utilities across all possible combinations of severity levels revealed a pattern of increasing marginal disutility from each additional unit of DLQI total score. This property, if confirmed by other studies, provides a rationale for prioritizing the treatment of patients with severe dermatological conditions over those with milder conditions, as this offers the greatest expected utility increase per unit reduction in DLQI score.

### Practical use and policy implications

Our proposed value set developed for DLQI health states (together with similar value sets to be obtained from follow-up studies) may have potentially wide applicability for the economic evaluation of dermatological interventions. In particular, it could be used for estimating the QALY impact of treatment options, which is a fundamental element in cost-utility analyses and hence is crucial for the efficient allocation of healthcare resources.

The study also has prospective implications for financing guidelines in dermatology. Our results confirm previous doubts about the DLQI [9, 31, 32], which raises concerns about its appropriateness as a benchmark in financing decisions. Efficiency and equity imply that access to healthcare interventions should be granted on the basis of cost-effectiveness analyses that use QALY improvements as an outcome measure. Nonetheless, in many European countries the criteria for reimbursement of dermatological treatments and medications are in terms of patients’ DLQI total scores [9], which would only be justified if the DLQI was homogeneous in terms of its impact on HRQoL. Yet, this appears far from being the case, as our regression results have pointed out substantial differences in the disutility impact of distinctive DLQI items.

As a consequence, health states with a given DLQI score can have a potentially wide range of different utility values depending on how the total score is broken down across DLQI items and severity levels. Likewise, a given reduction in DLQI score achieved by a dermatological treatment could correspond to substantially different amounts of QALY gains. This implies that cost-effectiveness analyses based on equally weighted DLQI scores are prone to be biased, which compromises the efficiency and equity of treatment allocation decisions. Therefore, we suggest that financing guidelines in dermatology should be reformed in a way to differentiate the HRQoL effects of different DLQI items and severity levels. This would require, in the first place, conducting confirmatory studies to verify the main tendencies implied by our proposed value set. Then, population-specific DLQI value sets could be developed through analyses similar to ours.

### Limitations and further research

Many of the methodological problems which we encountered may have been caused or exacerbated by specific aspects of the research design, such as the way in which the valuation task was set up and administered. These issues have implications for further research.

#### Online survey administration

Relying on internet-based methods for recruiting participants and administering the valuation task was presumably a primary cause of the poor quality of responses, even though it had obvious benefits in terms of low costs per respondent. Valuation data collected through crowdsourcing surveys is known to have questionable quality [33], so it would have been preferable to conduct the survey face-to-face, with the help of trained interviewers.

#### Construction of health states

A possible reason for the high proportion of individuals who gave identical or similar valuations may be the low diversity of health states in terms of their overall severity. The orthogonal design had as a consequence that three out of five health states in every random block had very similar DLQI total scores, which likely made it difficult for participants to differentiate across these health states in their valuations.

Therefore, it may have been better to use a different design, which would have resulted in a wider range of DLQI scores within each random block, and which would likely have facilitated better engagement of participants and induced greater variability in their valuations. In addition, using more random blocks could have strengthened the other aspect of diversity by providing a larger number of combinations as regards how the total score was broken down across the ten DLQI items.

#### TTO elicitation method

It should also be mentioned among the limitations that our chosen TTO utility elicitation method did not include a ‘worse than dead’ (WTD) task and neither did it use any complex iteration procedure for preference elicitation. These methodological choices were motivated by our concern about possible further deterioration of sample quality due to participants’ difficulty in task interpretation and/or their loss of interest, and consequently by our intent to reduce task complexity as much as possible. Besides, even with the inclusion of a complementary WTD task we would have expected to receive a low proportion of WTD responses [9]. (Indeed, the proportion of 0 utility valuations was less than 1% in our study.)

#### Comparison with other studies

Comparison of our results with other studies shows substantial differences. For example, in a similar study using a smaller set of health states [9] some of the mean TTO utility values were 0.10 to 0.15 lower than those predicted by our model.

#### Need for further research

For all the previous reasons, further research would be necessary to decide whether, in which way, and to what extent our results were affected by the quality of the sample, the chosen health states, and the survey administration method. This might involve replicating our study with an improved design, including the use of an enhanced set of health states and better methods for response elicitation.

For much the same reasons, our proposed value set should be considered as preliminary and it would need to be validated by follow-up studies before it can be applied in healthcare analysis and decision-making. Ideally, this would also involve verifying whether the main tendencies implied by our experimental value set are applicable to health state valuations made by individuals from relevant clinical populations.

## Conclusions

Our study is the first attempt to develop a societal value set for skin-related health states evaluated on the DLQI scale. Using the TTO valuation method, we have found substantial differences in the utility impact of distinctive DLQI items and severity levels. Our findings raise concerns about the current practice of defining treatment cost reimbursement criteria on the basis of equally weighted DLQI scores. Even though our value set is only preliminary and experimental, if corroborated by follow-up studies, it could be of considerable use in the economic evaluation of dermatological interventions as well as in the development of financing guidelines.

### Supplementary Information

**Additional file 1.** Assessment of health state utilities in dermatology: an experimental time trade-off value set for the dermatology life quality index.

## Data Availability

The data sets analyzed during the current study are openly available in the Mendeley Data repository, http://dx.doi.org/10.17632/f4r5by77wm.

## Appendix

### A.1 Dermatology life quality index questionnaire

***Symptoms and feelings***

[Q1] Over the last week, how itchy, sore, painful or stinging has your skin been?

[Q2] Over the last week, how embarrassed or self-conscious have you been because of your skin?

***Daily activities***

[Q3] Over the last week, how much has your skin interfered with you going shopping or looking after your home or garden?

[Q4] Over the last week, how much has your skin influenced the clothes you wear?

***Leisure***

[Q5] Over the last week, how much has your skin affected any social or leisure activities?

[Q6] Over the last week, how much has your skin made it difficult for you to do any sport?

***Work and school***

[Q7] Over the last week, has your skin prevented you from working or studying? If "No", over the last week, how much has your skin been a problem at work or studying?

***Personal relationships***

[Q8] Over the last week, how much has your skin created problems with your partner or any of your close friends or relatives?

[Q9] Over the last week, how much has your skin caused any sexual difficulties?

***Treatment***

[Q10] Over the last week, how much of a problem has the treatment for your skin been, for example, by making your home messy, or taking up time?

### A.2 Example time trade-off valuation task

Imagine there are two alternative lives to choose between: life ‘A’ (top green bar) and life ‘B’ (bottom blue bar). In life ‘B’ you have exactly 10 years to live but you suffer from a certain skin condition (see the description below). In life ‘A’ you live for a shorter period but in perfect health.

How many years of life ‘A’ (perfect health) would you deem equivalent to 10 years of life ‘B’ (skin condition)? Please indicate your answer by moving the mouse pointer over the top green bar.

***Life ‘B’—you live in the health state as follows:***

- Affects you very much:
  - Your skin affects your social or leisure activities very much.
  - You are very much embarrassed or self-conscious because of your skin.
- Affects you a lot:
  - Your skin creates a lot of problems with your partner or some of your close friends or relatives.
  - Your skin causes a lot sexual difficulties.
- Affects you a little:
  - Your skin is a little itchy, sore, painful or stinging.
- Does not affect you at all:
  - Your skin does not interfere with you at all going shopping or looking after your home or garden.
  - Your skin does not influence at all the clothes you wear.
  - Your skin does not make it difficult at all to do sports.
  - Your skin is not a problem at all at work or studying.
  - Treatment of your skin (for example by making your home messy, or by taking up time) is not a problem at all.

### A.3 Numerical example for the calculation of predicted utilities

We’ll calculate the predicted utility for health state ‘B’ described in the example valuation task (see Appendix A.2). State description ‘B’ translates to the following combination of DLQI severity levels: Q1:L1, Q2:L3, Q3:L0, Q4:L0, Q5:L3, Q6:L0, Q7:L0, Q8:L2, Q9:L2, Q10:L0. The calculation relies on the estimated partial effects and the regression intercept as below (see Table 4 in the main text).

Predicted utilities are obtained by adding to the intercept the cumulative disutilities associated with all non-zero severity levels (Q1:L1, Q2:L3, Q5:L3, Q8:L2, Q9:L2 in the current example):

- $${\hat{y}}$$ = 0.849 + (0.000—0.040—0.043—0.018—0.054) = 0.694 for traders;
- ${\hat{y}}_{a}$ = 0.873 + (0.000—0.034—0.036—0.015—0.046) = 0.742 for the total population.

The latter value concerning the total population can also be obtained by adjusting traders’ predicted utility for non-traders’ valuation according to (Eq. 6):

- $${\hat{y}}_{a}$$ = 0.158 × 1 + (1—0.158) × 0.694 = 0.742,

whereby *w* = 0.158 is the estimated proportion of non-traders within the total population.

## Abbreviations

DLQI: Dermatology Life Quality Index
TTO: Time Trade-Off
HRQoL: Health-Related Quality of Life
QALYs: Quality Adjusted Life Years
CV: Coefficient of variation
MAD: Mean Absolute Deviation
st. dev.: Standard deviation
lin. corr.: Linear correlation
coeff.: Coefficient
SE: Standard Error

## Footnotes

1 Originally a zero impact state with DLQI = 0 was included in the set of orthogonally designed health states, but we decided to replace it by a dermatologically more relevant health state with a score of 1 on the first DLQI item.

2 Non-traders were exempt from screening on response times because their resolute and supposedly predetermined behavior didn’t require lengthy deliberation about the number of life years to trade-off.

3 We considered other (logit, log–log) link functions as well as variable precision model versions but we found all of them were less stable and/or less robust (more prone to overfitting) than the basic model version.

4 A similar transformation was applied in [28]. However, their method was different from ours in two important respects: (1) their transformation was carried out on raw VAS (visual analogue scale) valuations rather than on TTO utilities; (2) their transformation wasn’t reversed like ours after fitting a regression model, i.e. they didn’t consider the distribution of effective scale ranges in calculating a social tariff for health states.
