# Supplementary material for: Assessment of health state utilities in dermatology: an experimental time trade-off value set for the dermatology life quality index
Source: Health Qual Life Outcomes. 2022 Jun 3;20:87. doi: 10.1186/s12955-022-01995-x (PMC9164408; doi:10.1186/s12955-022-01995-x)
Supplement: Supplementary file 1 — Additional file 1. Assessment of health state utilities in dermatology: an experimental time trade-off value set for the dermatology life quality index. [file 12955_2022_1995_MOESM1_ESM.pdf]

S.1 Supplement to exclusions by response time and response inconsistency

Exclusion by response time

Exclusion of participants due to too quick responses was considered in relation to a combination of two measures: the shortest ('min') and the median ('med') response time concerning the five valuations made by an individual. We experimented with several different combinations of exclusion thresholds: [thr_min] varying in the range {4, 5, …, 12 sec} and [thr_med] varying in the range {8, 9, …, 24 sec}.

We performed a 2×2 classification analysis on these two dimensions, aiming for a high degree of agreement between classification results by the two criteria, while trying to keep the number of exclusions at a reasonably low level. We used the F-score as a measure for the degree of agreement between the classification results on the two dimensions. The F-score was calculated as the harmonic mean between the 'precision' and 'recall' rates defined in the confusion matrix below.

Classification by criterion (2)	Classification by criterion (1)	
	exclude	don't exclude
exclude	n_{11}	n_{12}
don't exclude	n_{21}	n_{22}

precision: $P = n_{11} / (n_{11} + n_{12})$

recall: $R = n_{22} / (n_{21} + n_{22})$

F-score: $F = 2PR / (P + R)$

We plotted the F-scores against the number of joint exclusions, which was defined as the *intersection* of the two sets 'to be excluded'. We identified an 'efficient frontier' concerning the available (#exclusions; F-score) pairs, indicating the minimal number of exclusions necessary to reach a certain F-score (Fig. S1). In this way, the 9×17 initially considered combinations were narrowed down to 22 efficient combinations, from which the optimal one was to be selected subsequently.

Fig. S1 Decision boundary for sample restrictions based on minimal and median response times

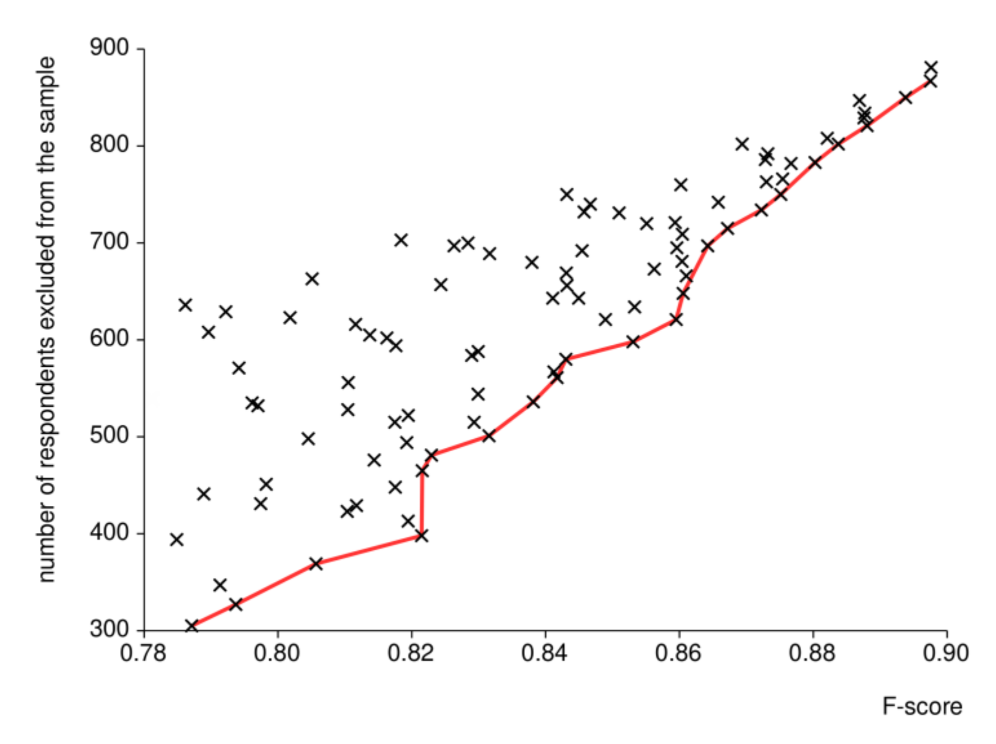

Exclusion by response inconsistency

As regards the maximal tolerable degree of inconsistency in participants' valuations, we imposed that all TTO utility differences with respect to the 'worst possible' health state (H73) must be greater than or equal to a certain threshold [thr_diff], which we varied in the range {-0.40, -0.35, ..., 0.00}. The optimal value of [thr_diff] was to be selected in the next step, conjointly with choosing the optimal value combination for the minimal and median response time thresholds.

To set the final values for the exclusion thresholds we performed another 2×2 classification analysis, whereby dimension (1) was the minimally required TTO utility difference with respect to H73, and dimension (2) comprised vectors of minimally required response times, which were allowed to vary within the previously identified range of efficient combinations. Again, we plotted the F-scores against the number of exclusions; however, joint exclusions were now defined by the *union* (rather than the intersection) of the two criteria, i.e. respondents were screened out in case either they gave too quick responses or their evaluations were too inconsistent.

Fig. S2 Decision boundary for sample restrictions based on response times and consistency of valuations

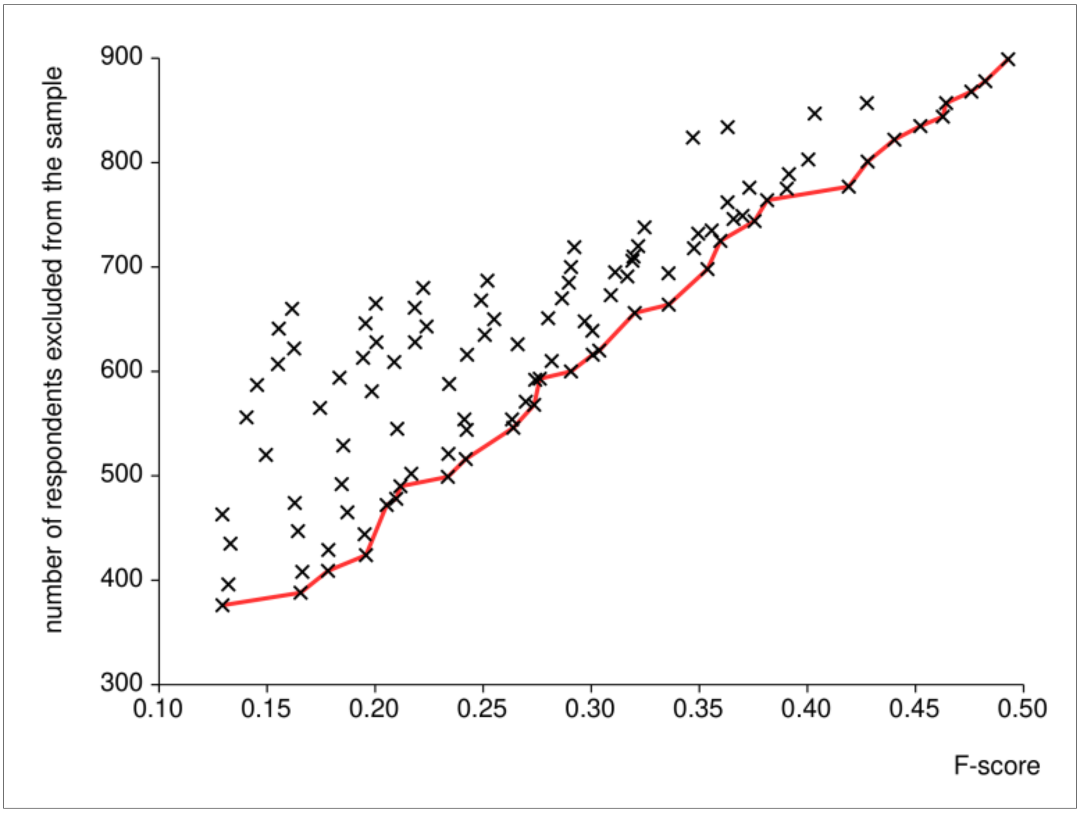

The efficient frontier concerning the set of (#exclusions; F-score) pairs was used as support for the final decision. Within the range of values eligible for the exclusion thresholds, the number of exclusions varied between 376 and 949, and the F-score varied between 0.129 and 0.554 (Fig. S2). We picked a reasonable-looking combination [thr_diff=(-0.10); thr_min=5; thr_med=10] around the middle of this region, resulting in 656 exclusions and an F-score of 0.320.

S.2 Supplement to the calculation of predicted utilities for traders

Combinations of DLQI severity levels (x) were mapped to predicted utility values $(\hat{y})$ by calculating weighted sums according to the corresponding vector of regression coefficients (β) and the regression intercept (α) , and (if applicable) applying the appropriate inverse link function to the linear predictor value thus obtained. This was carried out in different ways depending on the type of regression model.

1) In the case of ordinary linear regression models the usual scalar product formula was appropriate for calculating predicted utility values: $\hat{y} = \alpha + x'\beta$.
2) In the case of censored regression it was necessary to apply left- and right-censoring at the corresponding lower $(y_L=0)$ and upper $(y_U=1)$ thresholds: $\hat{y} = \max(\min(\alpha + x'\beta,\ 1),\ 0)$
3) In the case of ordinal regression we applied continuity correction in proportion to the relative position of the estimated latent variable value $(y^* = \alpha + x'\beta)$ between the lower (γ_L) and upper (γ_U) thresholds separating the predicted discrete TTO utility category $(\hat{y}_d)$ from the categories below $(\hat{y}_d - 0.05)$ and above $(\hat{y}_d + 0.05)$:
$\hat{y} = \hat{y}_d + 0.05(\alpha + x'\beta - (\gamma_U + \gamma_L)/2) / (\gamma_U + \gamma_L)$
4) In the case of beta regression the inverse link function $g^{-1}(\cdot)$ had to be applied to the linear predictor $\alpha + x'\beta$. By our choice of the probit link, $g^{-1}(\cdot)$ was equal to the standard normal cumulative distribution function $\Phi(\cdot)$ so that predicted utilities were obtained in the form $\hat{y} = \Phi(\alpha + x'\beta)$.
5) In the case of the two-part linear regression model first we applied the usual linear formula $(\hat{z} = \alpha + x'\beta)$ to estimate the relative disutility (z) from the health state (x) , which we further multiplied by the sample mean effective scale range $(\mathrm{m}(\lambda))$. Finally, the predicted utility was calculated by subtracting the scaled disutility from the utility of perfect health: $\hat{y} = 1 - \mathrm{m}(\lambda)\hat{z}$.
6) In the case of the two-part censored regression model we combined steps of the calculation as with models (2) and (5). First, the relative disutility (z) was estimated using the left-censored regression $\hat{z} = \max(\alpha + x'\beta, 0)$. Then, the predicted utility was obtained in the form $\hat{y} = 1 - \mathrm{m}(\lambda)\hat{z}$.
7) In the case of the two-part beta regression model we combined steps of the calculation as with models (4) and (5). First, the estimated relative disutility (z) was calculated by applying the inverse probit link function to the linear predictor: $\hat{z} = \Phi(\alpha + x'\beta)$. Then, the predicted utility was obtained in the form $\hat{y} = 1 - \mathrm{m}(\lambda)\hat{z}$.

S.3 Supplement to the effects of sample exclusions

The screening procedure was successful in enhancing the quality of the sample in terms of response times and consistency of valuations. Concerning the time taken to complete the valuation tasks, the sample mean was 14.8 seconds for the shortest response time across the five health states presented, whereas for the middle of the five response times the sample mean was 28.2 seconds. The overall sample mean concerning the average of the five response times was 36.5 seconds. For comparison: the same values for the initial sample were 9.4, 17.9, and 24.9 seconds, respectively.

Consistency of the valuations with respect to the 'worst possible' health state (H73) was significantly improved, as well. The mean difference between the TTO utility assigned to state H73 and the lowest of the four other valuations was -0.100, and the mean difference with respect to the average of the four other valuations was -0.215. For comparison: the same values for the initial sample were 0.045 (positive) and -0.047, respectively.

S.4 Supplement to cross-validation outcomes

Cross-validation (CV) was essential for eliminating model variables which did not have a consistently negative effect in every subsample (suppl. Tables S17, S18). CV fit indices improved monotonically along the model selection procedure (suppl. Tables S12–S14). Comparison between the initial, the intermediate, and the final model versions reveals that, in terms of average values across the seven types of models, linear correlation coefficients between the fitted and the observed mean utilities increased from 0.65 to 0.73 to 0.81, correlation with the observed medians increased from 0.58 to 0.64 to 0.74, and correlation with individual valuations increased from 0.34 to 0.35 to 0.36. Similarly, mean absolute differences (MAD) between the fitted and the observed mean utilities decreased (on average) from 0.044 to 0.038 to 0.032, MAD with respect to the observed medians decreased from 0.062 to 0.058 to 0.052, and MAD with respect to the individual valuations decreased from 0.200 to 0.199 to 0.198.

Supplementary tables

Table S1 Distribution of subjects by inclusion in the regression analysis

	Counts					Percentages				
	Excluded from sample	Retained			Total	Excluded from sample	Retained			Total
		non-trader	trader	all			non-trader	trader	all	
Total	1159	317	525	842	2001	57.9%	15.8%	26.2%	42.1%	100.0%

Table S2 Distribution of subjects by gender

	Counts					Column percentages					Adult pop. 2011
	Excluded from sample	Retained			Total	Excluded from sample	Retained			Total	
		non-trader	trader	all			non-trader	trader	all		
Female	623	144	313	457	1080	53.8%	45.4%	59.6%	54.3%	54.0%	53.4%
Male	536	173	212	385	921	46.2%	54.6%	40.4%	45.7%	46.0%	46.6%

Table S3 Distribution of subjects by age

	Counts					Column percentages					Adult pop. 2011
	Excluded from sample	Retained			Total	Excluded from sample	Retained			Total	
		non-trader	trader	all			non-trader	trader	all		
18-24	102	18	53	71	173	8.8%	5.7%	10.1%	8.4%	8.6%	10.5%
25-34	212	31	68	99	311	18.3%	9.8%	13.0%	11.8%	15.5%	16.9%
35-44	227	60	100	160	387	19.6%	18.9%	19.0%	19.0%	19.3%	18.8%
45-54	174	69	86	155	329	15.0%	21.8%	16.4%	18.4%	16.4%	15.5%
55-64	189	61	89	150	339	16.3%	19.2%	17.0%	17.8%	16.9%	17.6%
65+	255	78	129	207	462	22.0%	24.6%	24.6%	24.6%	23.1%	20.6%

Table S4 Distribution of subjects by place of residence

	Counts					Column percentages					Adult pop. 2011
	Excluded from sample	Retained			Total	Excluded from sample	Retained			Total	
		non-trader	trader	all			non-trader	trader	all		
Capital	211	54	96	150	361	18.2%	17.0%	18.3%	17.8%	18.0%	18.1%
City or major town	216	61	112	173	389	18.6%	19.2%	21.3%	20.5%	19.4%	17.9%
Minor town	424	107	181	288	712	36.6%	33.8%	34.5%	34.2%	35.6%	34.1%
Village	308	95	136	231	539	26.6%	30.0%	25.9%	27.4%	26.9%	30.0%

Table S5 Distribution of subjects by geographic region

	Counts					Column percentages					Adult pop. 2011
	Excluded from sample	Retained			Total	Excluded from sample	Retained			Total	
		non-trader	trader	all			non-trader	trader	all		
Central Hungary	334	95	167	262	596	28.8%	30.0%	31.8%	31.1%	29.8%	30.0%
Transdanubia	373	87	161	248	621	32.2%	27.4%	30.7%	29.5%	31.0%	30.4%
Great Plain and North	452	135	197	332	784	39.0%	42.6%	37.5%	39.4%	39.2%	39.6%

Table S6 Distribution of subjects by employment status

	Counts					Column percentages					Adult pop. 2011
	Excluded from sample	Retained			Total	Excluded from sample	Retained			Total	
		non-trader	trader	all			non-trader	trader	all		
Employed, full time	535	159	228	387	922	46.2%	50.2%	43.4%	46.0%	46.1%	48.2%
Employed, part time	75	15	23	38	113	6.5%	4.7%	4.4%	4.5%	5.6%	
Retired	273	82	141	223	496	23.6%	25.9%	26.9%	26.5%	24.8%	24.4%
Disability pensioner	56	10	28	38	94	4.8%	3.2%	5.3%	4.5%	4.7%	5.2%
Student	47	10	40	50	97	4.1%	3.2%	7.6%	5.9%	4.8%	5.5%
Unemployed	45	16	19	35	80	3.9%	5.0%	3.6%	4.2%	4.0%	6.9%
Out of labor force	10	2	3	5	15	0.9%	0.6%	0.6%	0.6%	0.7%	9.8%
Homemaker or housewife	76	18	14	32	108	6.6%	5.7%	2.7%	3.8%	5.4%	
Other	42	5	29	34	76	3.6%	1.6%	5.5%	4.0%	3.8%	

Table S7 Distribution of subjects by marital status

	Counts					Column percentages					Adult pop. 2011
	Excluded from sample	Retained			Total	Excluded from sample	Retained			Total	
		non-trader	trader	all			non-trader	trader	all		
Married	484	163	231	394	878	41.8%	51.4%	44.0%	46.8%	43.9%	47.7%
Divorced	117	43	61	104	221	10.1%	13.6%	11.6%	12.4%	11.0%	12.4%
Widowed	64	14	31	45	109	5.5%	4.4%	5.9%	5.3%	5.4%	12.3%
Domestic partnership	284	65	104	169	453	24.5%	20.5%	19.8%	20.1%	22.6%	27.5%
Single	210	32	98	130	340	18.1%	10.1%	18.7%	15.4%	17.0%	

Table S8 Distribution of subjects by level of education

	Counts					Column percentages					Adult pop. 2011
	Excluded from sample	Retained			Total	Excluded from sample	Retained			Total	
		non-trader	trader	all			non-trader	trader	all		
Primary school or less	77	17	20	37	114	6.6%	5.4%	3.8%	4.4%	5.7%	28.0%
Secondary school	888	232	388	620	1508	76.6%	73.2%	73.9%	73.6%	75.4%	53.1%
College or university	194	68	117	185	379	16.7%	21.5%	22.3%	22.0%	18.9%	18.8%

Table S9 Distribution of subjects by random block

Random block	Counts					Row percentages				
	Excluded from sample	Retained			Total	Excluded from sample	Retained			Total
		non-trader	trader	all			non-trader	trader	all	
B01	59	14	34	48	107	55.1%	13.1%	31.8%	44.9%	100.0%
B02	54	20	27	47	101	53.5%	19.8%	26.7%	46.5%	100.0%
B03	76	18	26	44	120	63.3%	15.0%	21.7%	36.7%	100.0%
B04	66	20	33	53	119	55.5%	16.8%	27.7%	44.5%	100.0%
B05	55	24	18	42	97	56.7%	24.7%	18.6%	43.3%	100.0%
B06	70	21	33	54	124	56.5%	16.9%	26.6%	43.5%	100.0%
B07	76	9	25	34	110	69.1%	8.2%	22.7%	30.9%	100.0%
B08	60	29	19	48	108	55.6%	26.9%	17.6%	44.4%	100.0%
B09	72	14	23	37	109	66.1%	12.8%	21.1%	33.9%	100.0%
B10	70	19	35	54	124	56.5%	15.3%	28.2%	43.5%	100.0%
B11	58	10	34	44	102	56.9%	9.8%	33.3%	43.1%	100.0%
B12	78	17	29	46	124	62.9%	13.7%	23.4%	37.1%	100.0%
B13	61	17	39	56	117	52.1%	14.5%	33.3%	47.9%	100.0%
B14	85	10	33	43	128	66.4%	7.8%	25.8%	33.6%	100.0%
B15	60	12	31	43	103	58.3%	11.7%	30.1%	41.7%	100.0%
B16	56	24	30	54	110	50.9%	21.8%	27.3%	49.1%	100.0%
B17	49	17	26	43	92	53.3%	18.5%	28.3%	46.7%	100.0%
B18	54	22	30	52	106	50.9%	20.8%	28.3%	49.1%	100.0%
all blocks	1159	317	525	842	2001	57.9%	15.8%	26.2%	42.1%	100.0%

Table S10 Characteristics of random blocks

Random block	Health states (from mildest to the most severe)					DLQI total score on each health state					DLQI total score on the five health states	
	#1	#2	#3	#4	#5	#1	#2	#3	#4	#5	mean	st. dev.
B01	H23	H13	H53	H56	H73	1	12	16	19	30	15.6	9.44
B02	H66	H04	H19	H07	H73	1	13	16	19	30	15.8	9.37
B03	H60	H31	H12	H45	H73	11	12	15	17	30	17.0	6.84
B04	H65	H40	H34	H15	H73	1	14	15	17	30	15.4	9.22
B05	H48	H58	H39	H06	H73	11	14	15	16	30	17.2	6.62
B06	H30	H63	H10	H22	H73	12	14	16	19	30	18.2	6.34
B07	H68	H59	H50	H38	H73	3	14	15	20	30	16.4	8.78
B08	H54	H57	H47	H11	H73	11	13	15	19	30	17.6	6.74
B09	H67	H61	H37	H29	H73	2	14	16	17	30	15.8	8.91
B10	H69	H18	H05	H14	H73	4	14	15	16	30	15.8	8.30
B11	H71	H20	H02	H42	H73	6	14	15	16	30	16.2	7.76
B12	H17	H26	H43	H55	H73	10	15	15	18	30	17.6	6.71
B13	H27	H03	H24	H09	H73	11	14	16	19	30	18.0	6.54
B14	H72	H64	H46	H36	H73	6	14	15	16	30	16.2	7.76
B15	H01	H62	H32	H16	H73	5	13	16	18	30	16.4	8.11
B16	H08	H21	H35	H44	H73	11	14	16	17	30	17.6	6.53
B17	H28	H33	H41	H25	H73	11	13	15	19	30	17.6	6.74
B18	H70	H52	H49	H51	H73	6	14	15	18	30	16.6	7.79

Table S11 Explanatory variables included in different model versions[a]

	1	2	3a	3b	3c	3d	3e	3f	3g	3h	3i	3j	3k	3l	3m	3n	3o	3p	3q	3r	3s	3t	3u	3v	3w	3x
D01_L1	X																									
D01_L2	X																									
D01_L3	X	X	X	X	X	X	X	X	X	X	X	X	X	X	X	X	X	X	X	X	X	X	X	X	X	X
D02_L1	X																									
D02_L2	X	X																								
D02_L3	X	X	X	X	X	X	X	X	X	X	X	X	X	X	X	X	X	X	X	X	X	X	X	X	X	X
D03_L1	X	X	X	X	X	X	X	X	X	X	X	X	X	X	X	X	X	X	X	X	X	X	X	X	X	X
D03_L2	X	X					X	X	X	X					X	X	X	X								
D03_L3	X	X	X	X	X	X					X	X	X	X												
D04_L1	X																									
D04_L2	X																									
D04_L3	X	X	X	X	X	X	X	X	X	X	X	X	X	X	X	X	X	X	X	X	X	X	X	X	X	X
D05_L1	X	X																								
D05_L2	X	X	X	X	X	X	X	X	X	X	X	X	X	X	X	X	X	X	X	X	X	X	X	X		
D05_L3	X	X	X	X	X	X	X	X	X	X	X	X	X	X	X	X	X	X	X	X	X	X	X	X	X	X
D06_L1	X	X									X		X		X		X		X		X					
D06_L2	X																									
D06_L3	X	X	X	X	X	X	X	X	X	X	X	X	X	X	X	X	X	X	X	X	X	X	X	X	X	X
D07_L1	X	X	X		X		X		X		X	X	X	X	X	X	X	X	X	X	X	X	X		X	
D07_L2	X	X	X	X	X	X	X	X	X	X	X	X	X	X	X	X	X	X	X	X	X	X	X	X	X	X
D07_L3	X	X	X	X	X	X	X	X	X	X	X	X	X	X	X	X	X	X	X	X	X	X	X	X	X	X
D08_L1	X	X																								
D08_L2	X	X	X	X	X	X	X	X	X	X	X	X	X	X	X	X	X	X	X	X	X	X	X	X	X	X
D08_L3	X	X	X	X	X	X	X	X	X	X	X	X	X	X	X	X	X	X	X	X	X	X	X	X	X	X
D09_L1	X	X	X	X	X	X	X	X	X	X	X	X	X	X	X	X	X	X	X	X	X	X	X	X	X	X
D09_L2	X	X	X	X	X	X	X	X	X	X	X	X	X	X	X	X	X	X	X	X	X	X	X	X	X	X
D09_L3	X	X	X	X			X	X			X	X	X	X	X	X	X	X	X	X	X	X				
D10_L1	X																									
D10_L2	X	X	X	X	X	X	X	X	X	X	X	X	X	X	X	X	X	X	X	X	X	X	X	X	X	X
D10_L3	X	X									X	X			X	X			X	X						

[a] Initial model: [1], intermediate model: [2], final model: [3x]

Table S12 Measures of cross-validation fit for the initial versions of the seven regression models

		linear	beta	censored	ordinal	scalable linear	scalable beta	scalable censored
linear correlation coefficient	w.r.t. individual TTO valuations	0.343	0.343	0.340	0.342	0.344	0.334	0.343
	w.r.t. mean TTO utilities	0.665	0.644	0.659	0.661	0.651	0.619	0.649
	w.r.t. median TTO utilities	0.597	0.590	0.594	0.592	0.580	0.542	0.578
mean absolute difference	w.r.t. individual TTO valuations	0.200	0.201	0.199	0.199	0.199	0.201	0.198
	w.r.t. mean TTO utilities	0.041	0.045	0.046	0.044	0.040	0.044	0.046
	w.r.t. median TTO utilities	0.067	0.061	0.063	0.062	0.063	0.059	0.060

Table S13 Measures of cross-validation fit for the intermediate versions of the seven regression models

		linear	beta	censored	ordinal	scalable linear	scalable beta	scalable censored
linear correlation coefficient	w.r.t. individual TTO valuations	0.353	0.353	0.352	0.353	0.354	0.345	0.354
	w.r.t. mean TTO utilities	0.739	0.718	0.738	0.738	0.729	0.691	0.731
	w.r.t. median TTO utilities	0.661	0.638	0.663	0.659	0.644	0.605	0.644
mean absolute difference	w.r.t. individual TTO valuations	0.199	0.200	0.199	0.198	0.198	0.201	0.197
	w.r.t. mean TTO utilities	0.034	0.038	0.039	0.039	0.035	0.041	0.040
	w.r.t. median TTO utilities	0.063	0.058	0.058	0.057	0.059	0.056	0.055

Table S14 Measures of cross-validation fit for the final versions of the seven regression models

		linear	beta	censored	ordinal	scalable linear	scalable beta	scalable censored
linear correlation coefficient	w.r.t. individual TTO valuations	0.363	0.361	0.361	0.362	0.365	0.360	0.364
	w.r.t. mean TTO utilities	0.815	0.793	0.808	0.813	0.825	0.808	0.823
	w.r.t. median TTO utilities	0.746	0.736	0.742	0.747	0.741	0.728	0.743
mean absolute difference	w.r.t. individual TTO valuations	0.198	0.199	0.197	0.197	0.197	0.200	0.196
	w.r.t. mean TTO utilities	0.029	0.034	0.033	0.033	0.029	0.035	0.034
	w.r.t. median TTO utilities	0.059	0.052	0.053	0.052	0.054	0.049	0.049

Table S15 Output for the seven types of regression models[a] (final versions)

	linear			beta			censored			ordinal		
	coeff[b]	SE	p-value	coeff[c]	SE	p-value	coeff[b]	SE	p-value	coeff[b]	SE	p-value
Intercept[d]	81.82	1.15	1.5E-56	82.56	NA	NA	84.96	1.24	9.6E-34	84.93	NA	NA
D01_L3	-1.96	0.95	0.039	-2.04	1.99	0.306	-1.69	1.14	0.136	-1.83	1.06	0.085
D02_L3	-3.83	0.74	2.7E-07	-3.55	1.40	0.011	-4.09	0.91	7.3E-06	-4.12	0.86	1.8E-06
D03_L1	-2.27	0.71	0.001	-2.06	1.19	0.085	-2.34	0.87	0.007	-2.64	0.82	0.001
D04_L3	-2.55	0.75	0.001	-3.51	1.47	0.017	-3.21	0.94	0.001	-3.00	0.88	6.7E-04
D05_L3	-3.39	0.75	6.8E-06	-5.29	1.40	1.6E-04	-3.98	0.93	1.8E-05	-3.96	0.88	7.3E-06
D06_L3	-2.47	0.74	0.001	-3.74	1.38	0.007	-2.82	0.91	0.002	-2.64	0.87	0.002
D07_L2	-2.29	0.82	0.005	-1.87	1.20	0.119	-2.72	0.96	0.005	-2.75	0.92	0.003
D07_L3	-1.88	0.99	0.057	-2.47	1.81	0.172	-2.17	1.17	0.065	-1.83	1.11	0.100
D08_L2	-1.18	0.82	0.148	-2.06	1.42	0.148	-1.72	1.01	0.089	-1.64	0.97	0.092
D08_L3	-3.10	0.98	0.002	-2.38	1.71	0.163	-3.01	1.18	0.011	-3.08	1.13	0.007
D09_L1	-3.14	0.88	3.9E-04	-3.12	1.41	0.027	-3.73	1.05	3.9E-04	-3.91	1.01	1.1E-04
D09_L2	-1.96	0.85	0.020	-1.84	1.42	0.197	-2.36	1.01	0.020	-1.98	0.97	0.041
D10_L2	-1.85	0.67	0.005	-0.91	1.15	0.432	-2.03	0.80	0.011	-2.01	0.77	0.009

[a] coefficients and standard errors in the table are in units of 0.01
[b] coefficients represent the incremental disutility with respect to the previous level of the DLQI item
[c] beta regression coefficients are rescaled in order to represent average partial effects
[d] p-values for the intercept are reported for the difference with respect to the value of 1

Table S15 (continued) Output for the seven types of regression models[a] (final versions)

	scalable linear			scalable beta			scalable censored		
	coeff[b]	SE	p-value	coeff[c]	SE	p-value	coeff[b]	SE	p-value
Intercept[d]	82.39	0.85	7.1E-96	82.29	NA	NA	84.82	0.88	3.1E-67
D01_L3	-2.84	0.86	0.001	-1.23	0.99	0.214	-2.73	0.91	0.003
D02_L3	-3.81	0.76	5.4E-07	-2.74	0.77	3.4E-04	-3.97	0.79	5.9E-07
D03_L1	-2.42	0.75	0.001	-1.66	0.69	0.017	-2.68	0.76	4.4E-04
D04_L3	-2.78	0.76	2.3E-04	-1.71	0.74	0.022	-3.07	0.81	1.6E-04
D05_L3	-4.12	0.77	8.5E-08	-4.16	0.77	6.5E-08	-4.57	0.81	1.5E-08
D06_L3	-2.03	0.75	0.007	-1.18	0.72	0.101	-2.18	0.81	0.007
D07_L2	-2.06	0.83	0.013	-1.25	0.82	0.127	-2.40	0.85	0.005
D07_L3	-1.71	0.97	0.078	-1.28	0.98	0.194	-1.64	1.02	0.106
D08_L2	-1.42	0.86	0.101	-1.39	0.86	0.109	-1.84	0.90	0.042
D08_L3	-3.15	1.00	0.002	-3.09	1.02	0.002	-3.07	1.04	0.003
D09_L1	-2.27	0.91	0.013	-2.25	0.86	0.009	-2.90	0.94	0.002
D09_L2	-1.89	0.86	0.028	-1.15	0.84	0.169	-1.85	0.89	0.039
D10_L2	-1.58	0.69	0.022	-1.69	0.69	0.015	-1.70	0.71	0.016

[a] coefficients and standard errors in the table are in units of 0.01
[b] coefficients represent the incremental disutility with respect to the previous level of the DLQI item
[c] beta regression coefficients are rescaled in order to represent average partial effects
[d] p-values for the intercept are reported for the difference with respect to the value of 1

Table S16 Cumulative partial effects estimated from the seven types of regression models[a] (final versions)

	Partial effects[b] for traders							Partial effects[b] adjusted for non-traders						
	linear	beta	cens.	ordin.	scal. linear	scal. beta	scal. cens.	linear	beta	cens.	ordin.	scal. linear	scal. beta	scal. cens.
Intercept	81.82	82.56	84.96	84.93	82.39	82.29	84.82	84.70	85.32	87.35	87.31	85.18	85.09	87.23
D01_L1<br>D01_L2	0.00	0.00	0.00	0.00	0.00	0.00	0.00	0.00	0.00	0.00	0.00	0.00	0.00	0.00
D01_L3	-1.96	-2.04	-1.69	-1.83	-2.84	-1.23	-2.73	-1.65	-1.72	-1.43	-1.54	-2.39	-1.04	-2.30
D02_L1<br>D02_L2	0.00	0.00	0.00	0.00	0.00	0.00	0.00	0.00	0.00	0.00	0.00	0.00	0.00	0.00
D02_L3	-3.83	-3.55	-4.09	-4.12	-3.81	-2.74	-3.97	-3.22	-2.99	-3.44	-3.46	-3.20	-2.30	-3.34
D03_L1<br>D03_L2<br>D03_L3	-2.27	-2.06	-2.34	-2.64	-2.42	-1.66	-2.68	-1.91	-1.73	-1.97	-2.22	-2.04	-1.39	-2.26
D04_L1<br>D04_L2	0.00	0.00	0.00	0.00	0.00	0.00	0.00	0.00	0.00	0.00	0.00	0.00	0.00	0.00
D04_L3	-2.55	-3.51	-3.21	-3.00	-2.78	-1.71	-3.07	-2.15	-2.95	-2.70	-2.53	-2.34	-1.44	-2.59
D05_L1<br>D05_L2	0.00	0.00	0.00	0.00	0.00	0.00	0.00	0.00	0.00	0.00	0.00	0.00	0.00	0.00
D05_L3	-3.39	-5.29	-3.98	-3.96	-4.12	-4.16	-4.57	-2.85	-4.46	-3.35	-3.33	-3.47	-3.50	-3.85
D06_L1<br>D06_L2	0.00	0.00	0.00	0.00	0.00	0.00	0.00	0.00	0.00	0.00	0.00	0.00	0.00	0.00
D06_L3	-2.47	-3.74	-2.82	-2.64	-2.03	-1.18	-2.18	-2.08	-3.14	-2.37	-2.22	-1.71	-0.99	-1.84
D07_L1	0.00	0.00	0.00	0.00	0.00	0.00	0.00	0.00	0.00	0.00	0.00	0.00	0.00	0.00
D07_L2	-2.29	-1.87	-2.72	-2.75	-2.06	-1.25	-2.40	-1.92	-1.58	-2.29	-2.32	-1.73	-1.05	-2.02
D07_L3	-4.17	-4.35	-4.88	-4.58	-3.77	-2.53	-4.04	-3.51	-3.66	-4.11	-3.86	-3.17	-2.13	-3.40
D08_L1	0.00	0.00	0.00	0.00	0.00	0.00	0.00	0.00	0.00	0.00	0.00	0.00	0.00	0.00
D08_L2	-1.18	-2.06	-1.72	-1.64	-1.42	-1.39	-1.84	-0.99	-1.74	-1.45	-1.38	-1.19	-1.17	-1.55
D08_L3	-4.28	-4.44	-4.73	-4.73	-4.57	-4.48	-4.91	-3.61	-3.74	-3.98	-3.98	-3.85	-3.77	-4.13
D09_L1	-3.14	-3.12	-3.73	-3.91	-2.27	-2.25	-2.90	-2.64	-2.62	-3.14	-3.29	-1.91	-1.90	-2.44
D09_L2<br>D09_L3	-5.10	-4.95	-6.09	-5.90	-4.17	-3.41	-4.74	-4.29	-4.17	-5.13	-4.96	-3.51	-2.87	-3.99
D10_L1	0.00	0.00	0.00	0.00	0.00	0.00	0.00	0.00	0.00	0.00	0.00	0.00	0.00	0.00
D10_L2<br>D10_L3	-1.85	-0.91	-2.03	-2.01	-1.58	-1.69	-1.70	-1.56	-0.76	-1.71	-1.69	-1.33	-1.42	-1.43

[a] figures in the table are in units of 0.01

[b] partial effects represent the cumulative disutility from distinctive levels of DLQI items

Table S17 Minimal and maximal regression coefficients[a] over 18 cross-validation subsamples concerning the initial versions of the seven regression models[b]

	Min							Max						
	linear	beta	cens.	ordin.	scal. linear	scal. beta	scal. cens.	linear	beta	cens.	ordin.	scal. linear	scal. beta	scal. cens.
D01_L1	-1.08	-0.05	-0.57	-1.13	-0.57	-0.80	-0.46	0.70	2.70	1.64	1.17	0.91	0.78	1.38
D01_L2	-0.80	-0.04	-0.63	-0.87	-0.99	-0.67	-1.05	0.73	1.28	0.59	0.76	0.31	0.25	0.20
D01_L3	-3.31	-3.66	-3.10	-3.14	-3.30	-1.49	-3.24	-1.08	-2.35	-0.87	-0.84	-1.76	-0.61	-1.65
D02_L1	-1.86	-1.19	-2.57	-2.03	-1.39	-1.01	-1.50	0.82	1.47	1.17	1.10	0.75	0.82	1.00
D02_L2	-1.28	-3.16	-2.05	-1.66	-1.57	-1.82	-2.09	0.61	-0.77	0.41	0.49	0.24	-0.33	0.06
D02_L3	-4.09	-3.63	-4.35	-4.31	-4.02	-2.84	-4.12	-2.43	-0.78	-2.02	-2.30	-1.82	-0.72	-1.65
D03_L1	-2.08	-3.08	-1.56	-2.39	-2.49	-2.37	-2.80	-0.48	-0.87	0.34	-0.43	-0.93	-0.65	-0.89
D03_L2	-1.29	-0.81	-2.60	-1.68	-0.81	-0.21	-1.11	0.15	1.38	-0.90	0.05	0.65	1.49	0.74
D03_L3	-1.51	-1.83	-0.91	-1.61	-2.56	-2.37	-2.59	-0.05	-0.52	0.82	0.02	-1.18	-1.25	-1.12
D04_L1	-1.03	-0.50	-1.60	-1.08	-0.60	-0.75	-0.85	0.58	1.53	0.44	0.70	1.01	0.65	1.16
D04_L2	-0.54	-0.25	-0.25	-0.48	-0.09	0.22	-0.09	0.90	2.14	1.27	1.34	1.68	2.26	1.99
D04_L3	-3.81	-4.96	-4.86	-4.77	-4.00	-2.89	-4.56	-0.80	-2.59	-1.27	-1.28	-2.05	-0.86	-2.27
D05_L1	-0.90	-0.76	-1.65	-1.14	-1.01	0.03	-1.29	0.51	0.50	0.14	0.37	0.17	1.10	0.13
D05_L2	-2.05	-0.36	-1.85	-2.25	-1.60	-1.46	-1.77	-0.40	1.29	-0.07	-0.39	0.09	0.12	0.09
D05_L3	-3.67	-6.43	-4.32	-4.28	-4.49	-4.62	-4.90	-1.60	-4.59	-2.22	-2.14	-3.01	-3.33	-3.35
D06_L1	-1.58	-1.59	-1.80	-1.78	-0.30	-0.37	-0.56	0.03	-0.17	-0.18	0.04	0.81	0.66	0.62
D06_L2	-0.32	0.18	-1.02	-0.44	-0.45	-0.10	-0.49	0.95	2.04	0.65	0.96	0.62	0.88	0.72
D06_L3	-2.76	-4.26	-3.25	-3.05	-2.37	-1.37	-2.58	-1.41	-2.48	-1.13	-1.33	-0.75	0.07	-0.72
D07_L1	-1.92	-2.24	-2.26	-2.58	-1.74	-1.27	-2.22	-0.16	-0.67	-0.28	-0.50	-0.48	-0.33	-0.79
D07_L2	-2.70	-2.10	-2.91	-3.06	-2.51	-1.73	-2.78	-1.52	-0.65	-1.52	-1.65	-1.11	-0.47	-1.05
D07_L3	-2.40	-3.26	-3.13	-2.33	-1.98	-1.66	-1.90	-0.30	-1.90	-0.47	-0.02	-0.46	-0.65	-0.33
D08_L1	-1.40	-1.95	-2.08	-1.72	-0.65	-0.06	-0.92	0.14	-0.19	-0.06	0.02	0.84	1.07	0.71
D08_L2	-1.34	-2.80	-1.89	-1.90	-2.63	-2.99	-3.04	0.53	-0.47	0.44	0.43	-0.71	-1.21	-0.89
D08_L3	-4.12	-3.47	-4.35	-4.29	-3.97	-3.46	-4.02	-2.09	-1.48	-1.80	-2.04	-2.17	-1.74	-2.00
D09_L1	-3.93	-3.95	-4.33	-4.92	-3.50	-3.53	-3.96	-2.37	-1.65	-2.58	-2.98	-1.83	-1.64	-2.17
D09_L2	-3.35	-2.68	-4.37	-3.48	-2.08	-1.09	-2.16	-0.40	0.10	-1.12	-0.13	0.10	0.43	0.34
D09_L3	-1.87	-2.58	-1.34	-2.00	-1.95	-2.02	-2.06	0.40	-0.22	1.10	0.46	0.16	-0.01	0.28
D10_L1	-0.03	-0.99	0.58	0.06	0.81	0.20	0.80	1.62	1.06	2.75	1.95	2.43	1.53	2.58
D10_L2	-2.53	-1.54	-3.38	-2.83	-2.31	-1.79	-2.49	-0.73	0.25	-1.40	-0.87	-0.84	-0.71	-0.92
D10_L3	-1.10	-1.17	-1.11	-1.28	-2.37	-2.35	-2.53	0.14	0.45	0.24	0.10	-1.09	-1.25	-1.07

[a] coefficients represent the incremental disutility with respect to the previous level of the DLQI item
[b] figures in the table are in units of 0.01

Table S18 Minimal and maximal regression coefficients[a] over 18 cross-validation subsamples concerning the final versions of the seven regression models[b]

	Min							Max						
	linear	beta	cens.	ordin.	scal. linear	scal. beta	scal. cens.	linear	beta	cens.	ordin.	scal. linear	scal. beta	scal. cens.
D01_L1														
D01_L2														
D01_L3	-2.63	-2.69	-2.29	-2.50	-3.44	-1.90	-3.31	-1.34	-1.61	-0.93	-1.12	-2.45	-0.69	-2.27
D02_L1														
D02_L2														
D02_L3	-4.25	-4.36	-4.65	-4.60	-4.44	-3.35	-4.65	-3.42	-2.73	-3.60	-3.65	-3.43	-2.29	-3.54
D03_L1	-2.62	-2.48	-2.73	-3.05	-2.78	-2.01	-3.07	-1.59	-1.75	-1.59	-1.90	-1.84	-1.25	-2.08
D03_L2														
D03_L3														
D04_L1														
D04_L2														
D04_L3	-3.11	-4.00	-3.84	-3.59	-3.22	-2.16	-3.54	-1.32	-2.32	-1.82	-1.67	-1.69	-0.97	-1.90
D05_L1														
D05_L2														
D05_L3	-4.14	-5.93	-4.82	-4.74	-4.69	-4.63	-5.16	-2.96	-4.71	-3.49	-3.50	-3.62	-3.65	-4.01
D06_L1														
D06_L2														
D06_L3	-2.81	-4.15	-3.26	-2.97	-2.30	-1.47	-2.48	-1.96	-2.85	-2.26	-2.09	-1.59	-0.68	-1.69
D07_L1														
D07_L2	-2.67	-2.26	-3.13	-3.20	-2.51	-1.68	-2.89	-1.73	-1.44	-2.19	-2.19	-1.48	-0.64	-1.76
D07_L3	-2.45	-3.60	-2.77	-2.36	-2.08	-1.77	-2.09	-1.25	-2.09	-1.59	-1.20	-1.31	-0.81	-1.24
D08_L1														
D08_L2	-1.66	-2.79	-2.31	-2.21	-2.10	-2.11	-2.56	-0.58	-1.54	-1.10	-1.06	-0.88	-0.92	-1.33
D08_L3	-3.62	-2.99	-3.59	-3.63	-3.78	-3.70	-3.78	-2.50	-1.59	-2.31	-2.40	-2.53	-2.24	-2.35
D09_L1	-3.89	-3.82	-4.49	-4.62	-3.03	-2.97	-3.60	-2.77	-2.59	-3.32	-3.48	-1.90	-1.66	-2.47
D09_L2	-2.90	-2.74	-3.43	-3.03	-2.71	-1.68	-2.76	-1.59	-1.15	-2.02	-1.62	-1.36	-0.65	-1.21
D09_L3														
D10_L1														
D10_L2	-2.29	-1.77	-2.53	-2.53	-1.96	-2.06	-2.16	-1.52	-0.43	-1.67	-1.67	-1.13	-1.24	-1.21
D10_L3														

[a] coefficients represent the incremental disutility with respect to the previous level of the DLQI item
[b] figures in the table are in units of 0.01

Table S19 Health state characteristics and valuations by ‘trader’ subjects

Health state	DLQI profile	DLQI sum	n	Mean utility	Percentiles of TTO utility						
					5%	10%	25%	50%	75%	90%	95%
H01	2100100101	5	31	0.792	0.203	0.480	0.703	0.863	0.963	1.000	1.000
H02	2110330131	15	34	0.687	0.260	0.385	0.517	0.725	0.883	0.998	1.000
H03	2031320102	14	39	0.691	0.199	0.270	0.506	0.717	0.918	1.000	1.000
H04	0132301210	13	27	0.720	0.479	0.492	0.588	0.713	0.891	0.941	0.958
H05	2103021312	15	35	0.691	0.163	0.350	0.492	0.794	0.929	1.000	1.000
H06	1302013231	16	18	0.711	0.170	0.415	0.581	0.700	0.917	0.983	1.000
H07	0332112313	19	27	0.704	0.293	0.484	0.583	0.700	0.881	0.983	1.000
H08	3001221020	11	30	0.743	0.350	0.475	0.650	0.789	0.900	1.000	1.000
H09	3101313322	19	39	0.626	0.108	0.198	0.488	0.683	0.808	1.000	1.000
H10	1202121133	16	33	0.727	0.108	0.383	0.538	0.810	0.940	0.981	0.995
H11	2303232211	19	19	0.684	0.173	0.298	0.519	0.713	0.881	1.000	1.000
H12	0021122331	15	26	0.692	0.333	0.365	0.500	0.775	0.917	0.968	0.984
H13	0100132302	12	34	0.682	0.160	0.360	0.580	0.700	0.850	0.970	0.991
H14	2331001303	16	35	0.679	0.069	0.113	0.509	0.790	0.894	1.000	1.000
H15	1123322003	17	33	0.714	0.303	0.340	0.503	0.793	0.922	0.984	1.000
H16	3133120230	18	31	0.707	0.303	0.378	0.509	0.763	0.881	1.000	1.000
H17	1111111111	10	29	0.817	0.298	0.598	0.713	0.894	1.000	1.000	1.000
H18	1211230310	14	35	0.689	0.104	0.300	0.505	0.783	0.905	0.967	1.000
H19	0013311223	16	27	0.683	0.384	0.418	0.522	0.706	0.831	0.913	0.958
H20	1311302012	14	34	0.629	0.203	0.245	0.486	0.625	0.850	0.952	0.980
H21	3220110212	14	30	0.700	0.388	0.475	0.513	0.713	0.850	0.975	1.000
H22	1330220323	19	33	0.658	0.108	0.283	0.406	0.688	0.954	1.000	1.000
H23	1000000000	1	34	0.844	0.318	0.635	0.779	0.925	1.000	1.000	1.000
H24	2122103023	16	39	0.683	0.123	0.207	0.413	0.756	1.000	1.000	1.000
H25	3212323300	19	26	0.675	0.083	0.115	0.500	0.725	0.956	1.000	1.000
H26	1230312021	15	29	0.679	0.298	0.398	0.503	0.733	0.883	0.930	1.000
H27	2003113010	11	39	0.659	0.123	0.290	0.484	0.700	0.881	1.000	1.000
H28	3112002101	11	26	0.700	0.040	0.090	0.513	0.875	0.981	1.000	1.000
H29	2310123032	17	23	0.644	0.133	0.283	0.493	0.688	0.888	1.000	1.000
H30	0300321201	12	33	0.761	0.203	0.390	0.588	0.821	0.956	0.998	1.000
H31	0200213103	12	26	0.704	0.308	0.355	0.538	0.725	0.883	1.000	1.000
H32	2231133001	16	31	0.769	0.403	0.493	0.654	0.796	0.918	1.000	1.000
H33	3301100221	13	26	0.660	0.040	0.090	0.500	0.675	0.950	1.000	1.000
H34	0321203130	15	33	0.655	0.208	0.290	0.498	0.663	0.894	0.987	1.000
H35	2322310120	16	30	0.687	0.350	0.482	0.514	0.708	0.821	1.000	1.000
H36	3120231013	16	33	0.736	0.416	0.480	0.578	0.767	0.897	1.000	1.000
H37	2022031321	16	23	0.694	0.133	0.240	0.503	0.788	0.946	1.000	1.000

Table S19 (continued) Health state characteristics and valuations by ‘trader’ subjects

Health state	DLQI profile	DLQI sum	n	Mean utility	Percentiles of TTO utility						
					5%	10%	25%	50%	75%	90%	95%
H38	2222222222	20	25	0.656	0.231	0.263	0.488	0.700	0.884	1.000	1.000
H39	1130033220	15	18	0.658	0.170	0.485	0.519	0.615	0.813	0.985	1.000
H40	0213102320	14	33	0.673	0.291	0.390	0.505	0.713	0.823	0.968	1.000
H41	3312211002	15	26	0.706	0.108	0.155	0.400	0.875	0.981	1.000	1.000
H42	3020303311	16	34	0.606	0.210	0.345	0.486	0.575	0.788	0.915	1.000
H43	2010202233	15	29	0.747	0.348	0.490	0.563	0.813	0.906	1.000	1.000
H44	3201032123	17	30	0.685	0.338	0.475	0.520	0.695	0.820	1.000	1.000
H45	0032233112	17	26	0.698	0.190	0.340	0.500	0.785	0.900	1.000	1.000
H46	2203300113	15	33	0.808	0.458	0.580	0.731	0.850	0.923	0.988	1.000
H47	1323131100	15	19	0.687	0.223	0.320	0.519	0.717	0.856	1.000	1.000
H48	0232020011	11	18	0.706	0.170	0.415	0.581	0.700	0.913	0.980	1.000
H49	3012130203	15	30	0.693	0.213	0.400	0.538	0.700	0.888	1.000	1.000
H50	1223003202	15	25	0.710	0.288	0.350	0.513	0.750	0.921	0.983	1.000
H51	3233201031	18	30	0.698	0.213	0.400	0.519	0.725	0.933	1.000	1.000
H52	3320022110	14	30	0.690	0.300	0.350	0.490	0.725	0.938	1.000	1.000
H53	0113223121	16	34	0.631	0.160	0.285	0.480	0.675	0.821	0.890	0.940
H54	1030101122	11	19	0.737	0.273	0.398	0.588	0.788	0.913	1.000	1.000
H55	3033012132	18	29	0.686	0.198	0.398	0.513	0.717	0.888	0.979	1.000
H56	0221331232	19	34	0.629	0.093	0.195	0.500	0.688	0.820	0.901	0.918
H57	1023210301	13	19	0.729	0.173	0.320	0.613	0.720	1.000	1.000	1.000
H58	0313030022	14	18	0.689	0.170	0.488	0.583	0.675	0.850	0.980	1.000
H59	1011023213	14	25	0.692	0.088	0.400	0.503	0.783	0.919	0.983	1.000
H60	0121010033	11	26	0.723	0.383	0.415	0.519	0.775	0.913	1.000	1.000
H61	1102200332	14	23	0.670	0.183	0.433	0.497	0.619	1.000	1.000	1.000
H62	2210011330	13	31	0.677	0.203	0.328	0.519	0.710	0.878	1.000	1.000
H63	1002332030	14	33	0.682	0.108	0.333	0.479	0.713	0.943	0.992	1.000
H64	2131212200	14	33	0.786	0.408	0.508	0.678	0.830	0.922	1.000	1.000
H65	0100000000	1	33	0.867	0.503	0.540	0.806	0.930	1.000	1.000	1.000
H66	0001000000	1	27	0.817	0.484	0.518	0.763	0.821	0.996	1.000	1.000
H67	1100000000	2	23	0.804	0.383	0.440	0.663	0.888	0.996	1.000	1.000
H68	1001000001	3	25	0.818	0.481	0.513	0.688	0.910	0.987	1.000	1.000
H69	1110100000	4	35	0.827	0.363	0.494	0.744	0.906	1.000	1.000	1.000
H70	3300000000	6	30	0.722	0.300	0.375	0.517	0.800	0.906	1.000	1.000
H71	1110111000	6	34	0.765	0.480	0.492	0.600	0.792	0.933	1.000	1.000
H72	2020002000	6	33	0.827	0.508	0.590	0.738	0.850	0.971	1.000	1.000
H73	3333333333	30	525	0.496	0.033	0.107	0.317	0.505	0.690	0.824	0.912

Table S20 Valuation of health states by 'trader' subjects, in comparison with fitted values from the seven types of regression models

Health state	Observed TTO utilities					Fitted utilities						
	mean	median	st. dev.	min	max	linear	beta	cens.	ordin.	scaled linear	scaled beta	scaled cens.
H01	0.792	0.863	0.232	0.050	1.000	0.818	0.826	0.850	0.849	0.824	0.823	0.848
H02	0.687	0.725	0.238	0.050	1.000	0.686	0.680	0.697	0.698	0.697	0.710	0.706
H03	0.691	0.717	0.270	0.100	1.000	0.743	0.757	0.766	0.763	0.743	0.742	0.759
H04	0.720	0.713	0.173	0.400	0.950	0.719	0.716	0.732	0.728	0.722	0.720	0.728
H05	0.691	0.794	0.273	0.100	1.000	0.700	0.722	0.713	0.713	0.712	0.713	0.722
H06	0.711	0.700	0.237	0.150	1.000	0.675	0.692	0.682	0.687	0.692	0.713	0.702
H07	0.704	0.700	0.212	0.200	1.000	0.642	0.681	0.653	0.648	0.657	0.671	0.663
H08	0.743	0.789	0.212	0.150	1.000	0.748	0.768	0.772	0.772	0.754	0.773	0.773
H09	0.626	0.683	0.270	0.100	1.000	0.611	0.616	0.616	0.619	0.613	0.637	0.621
H10	0.727	0.810	0.274	0.000	1.000	0.749	0.778	0.768	0.770	0.767	0.768	0.784
H11	0.684	0.713	0.248	0.150	1.000	0.664	0.661	0.667	0.669	0.680	0.708	0.685
H12	0.692	0.775	0.237	0.200	1.000	0.679	0.708	0.691	0.689	0.692	0.705	0.701
H13	0.682	0.700	0.233	0.100	1.000	0.709	0.731	0.727	0.728	0.722	0.729	0.736
H14	0.679	0.790	0.283	0.050	1.000	0.696	0.731	0.718	0.714	0.700	0.708	0.716
H15	0.714	0.793	0.238	0.300	1.000	0.695	0.705	0.707	0.706	0.694	0.709	0.704
H16	0.707	0.763	0.228	0.200	1.000	0.688	0.695	0.699	0.699	0.688	0.721	0.698
H17	0.817	0.894	0.230	0.000	1.000	0.764	0.784	0.789	0.784	0.777	0.781	0.792
H18	0.689	0.783	0.267	0.100	1.000	0.697	0.708	0.713	0.710	0.711	0.719	0.722
H19	0.683	0.706	0.196	0.200	1.000	0.655	0.651	0.656	0.658	0.659	0.671	0.662
H20	0.629	0.625	0.247	0.200	1.000	0.651	0.672	0.661	0.655	0.661	0.674	0.666
H21	0.700	0.713	0.206	0.200	1.000	0.714	0.739	0.735	0.729	0.719	0.734	0.730
H22	0.658	0.688	0.300	0.000	1.000	0.645	0.682	0.657	0.655	0.658	0.672	0.668
H23	0.844	0.925	0.192	0.300	1.000	0.818	0.826	0.850	0.849	0.824	0.823	0.848
H24	0.683	0.756	0.304	0.050	1.000	0.684	0.719	0.696	0.698	0.705	0.722	0.717
H25	0.675	0.725	0.310	0.050	1.000	0.658	0.658	0.673	0.672	0.647	0.671	0.659
H26	0.679	0.733	0.240	0.000	1.000	0.688	0.700	0.698	0.697	0.696	0.709	0.704
H27	0.659	0.700	0.269	0.050	1.000	0.720	0.731	0.731	0.734	0.736	0.753	0.748
H28	0.700	0.875	0.334	0.000	1.000	0.753	0.777	0.782	0.777	0.751	0.779	0.770
H29	0.644	0.688	0.273	0.100	1.000	0.646	0.683	0.655	0.657	0.667	0.692	0.677
H30	0.761	0.821	0.248	0.200	1.000	0.734	0.732	0.752	0.752	0.730	0.733	0.744
H31	0.704	0.725	0.214	0.300	1.000	0.758	0.783	0.781	0.783	0.770	0.778	0.791
H32	0.769	0.796	0.186	0.350	1.000	0.729	0.739	0.749	0.751	0.742	0.765	0.759
H33	0.660	0.675	0.321	0.000	1.000	0.698	0.715	0.714	0.714	0.702	0.728	0.715
H34	0.655	0.663	0.257	0.050	1.000	0.665	0.692	0.676	0.677	0.682	0.710	0.694
H35	0.687	0.708	0.228	0.000	1.000	0.672	0.682	0.685	0.683	0.679	0.693	0.689
H36	0.736	0.767	0.191	0.400	1.000	0.701	0.723	0.724	0.719	0.712	0.736	0.726
H37	0.694	0.788	0.279	0.100	1.000	0.677	0.689	0.690	0.690	0.692	0.706	0.703

Table S20 (continued) Valuation of health states by ‘trader’ subjects, in comparison with fitted values from the seven types of regression models

Health state	Observed TTO utilities					Fitted utilities						
	mean	median	st. dev.	min	max	linear	beta	cens.	ordin.	scaled linear	scaled beta	scaled cens.
H38	0.656	0.700	0.263	0.100	1.000	0.691	0.723	0.701	0.700	0.708	0.721	0.715
H39	0.658	0.615	0.213	0.150	1.000	0.666	0.669	0.671	0.675	0.686	0.712	0.693
H40	0.673	0.713	0.233	0.050	1.000	0.653	0.672	0.659	0.659	0.664	0.687	0.670
H41	0.706	0.875	0.328	0.100	1.000	0.719	0.754	0.748	0.743	0.717	0.744	0.737
H42	0.606	0.575	0.230	0.000	1.000	0.626	0.624	0.636	0.633	0.624	0.648	0.630
H43	0.747	0.813	0.206	0.300	1.000	0.691	0.723	0.701	0.700	0.708	0.721	0.715
H44	0.685	0.695	0.234	0.000	1.000	0.682	0.706	0.696	0.698	0.697	0.728	0.711
H45	0.698	0.785	0.264	0.050	1.000	0.679	0.700	0.692	0.691	0.703	0.722	0.713
H46	0.808	0.850	0.163	0.400	1.000	0.709	0.713	0.720	0.720	0.716	0.716	0.726
H47	0.687	0.717	0.230	0.200	1.000	0.707	0.713	0.725	0.725	0.714	0.744	0.729
H48	0.706	0.700	0.234	0.150	1.000	0.764	0.784	0.789	0.784	0.777	0.781	0.792
H49	0.693	0.700	0.232	0.200	1.000	0.721	0.733	0.744	0.742	0.721	0.746	0.737
H50	0.710	0.750	0.246	0.150	1.000	0.698	0.713	0.708	0.711	0.704	0.725	0.715
H51	0.698	0.725	0.235	0.200	1.000	0.699	0.716	0.716	0.716	0.702	0.736	0.716
H52	0.690	0.725	0.248	0.200	1.000	0.683	0.715	0.704	0.697	0.690	0.724	0.702
H53	0.631	0.675	0.239	0.100	1.000	0.677	0.693	0.684	0.688	0.693	0.722	0.703
H54	0.737	0.788	0.228	0.250	1.000	0.726	0.760	0.745	0.744	0.742	0.750	0.757
H55	0.686	0.717	0.248	0.000	1.000	0.658	0.688	0.669	0.668	0.665	0.704	0.675
H56	0.629	0.688	0.256	0.050	1.000	0.656	0.649	0.660	0.661	0.667	0.677	0.671
H57	0.729	0.720	0.238	0.150	1.000	0.727	0.741	0.747	0.746	0.726	0.738	0.742
H58	0.689	0.675	0.216	0.150	1.000	0.637	0.652	0.644	0.646	0.656	0.688	0.665
H59	0.692	0.783	0.268	0.050	1.000	0.692	0.716	0.703	0.701	0.709	0.719	0.717
H60	0.723	0.775	0.223	0.200	1.000	0.726	0.760	0.745	0.744	0.742	0.750	0.757
H61	0.670	0.619	0.254	0.150	1.000	0.706	0.738	0.721	0.723	0.721	0.719	0.735
H62	0.677	0.710	0.252	0.000	1.000	0.702	0.727	0.718	0.717	0.712	0.719	0.725
H63	0.682	0.713	0.288	0.000	1.000	0.686	0.682	0.694	0.697	0.700	0.714	0.709
H64	0.786	0.830	0.184	0.300	1.000	0.761	0.777	0.782	0.779	0.765	0.777	0.779
H65	0.867	0.930	0.169	0.500	1.000	0.818	0.826	0.850	0.849	0.824	0.823	0.848
H66	0.817	0.821	0.196	0.200	1.000	0.818	0.826	0.850	0.849	0.824	0.823	0.848
H67	0.804	0.888	0.226	0.250	1.000	0.818	0.826	0.850	0.849	0.824	0.823	0.848
H68	0.818	0.910	0.225	0.100	1.000	0.818	0.826	0.850	0.849	0.824	0.823	0.848
H69	0.827	0.906	0.207	0.300	1.000	0.796	0.810	0.826	0.823	0.800	0.806	0.821
H70	0.722	0.800	0.250	0.000	1.000	0.760	0.781	0.792	0.790	0.757	0.781	0.781
H71	0.765	0.792	0.190	0.400	1.000	0.796	0.810	0.826	0.823	0.800	0.806	0.821
H72	0.827	0.850	0.168	0.300	1.000	0.773	0.795	0.799	0.795	0.779	0.792	0.797
H73	0.496	0.505	0.252	0.000	0.950	0.499	0.468	0.491	0.495	0.503	0.578	0.502

Table S21 Valuation of health states adjusted for non-traders, in comparison with fitted values from the seven types of regression models

Health state	Observed TTO		Fitted utilities						
	mean	median	linear	beta	cens.	ordin.	scaled linear	scaled beta	scaled cens.
H01	0.825	0.899	0.847	0.853	0.874	0.873	0.852	0.851	0.872
H02	0.736	0.790	0.736	0.731	0.745	0.746	0.745	0.756	0.753
H03	0.740	0.815	0.784	0.795	0.803	0.801	0.784	0.783	0.797
H04	0.765	0.792	0.763	0.761	0.774	0.771	0.766	0.764	0.771
H05	0.740	0.865	0.748	0.766	0.758	0.758	0.758	0.758	0.766
H06	0.757	0.860	0.727	0.741	0.732	0.737	0.741	0.759	0.750
H07	0.751	0.788	0.698	0.732	0.708	0.704	0.711	0.723	0.716
H08	0.784	0.810	0.788	0.805	0.808	0.808	0.793	0.809	0.809
H09	0.685	0.754	0.672	0.677	0.676	0.680	0.675	0.694	0.681
H10	0.771	0.891	0.789	0.813	0.805	0.807	0.804	0.805	0.818
H11	0.734	0.791	0.717	0.715	0.720	0.721	0.731	0.755	0.735
H12	0.741	0.806	0.730	0.754	0.740	0.738	0.741	0.752	0.748
H13	0.733	0.755	0.755	0.774	0.770	0.771	0.766	0.772	0.778
H14	0.730	0.823	0.744	0.774	0.763	0.760	0.748	0.754	0.761
H15	0.759	0.815	0.743	0.752	0.753	0.752	0.743	0.755	0.751
H16	0.753	0.795	0.737	0.743	0.747	0.747	0.737	0.765	0.745
H17	0.846	0.929	0.802	0.818	0.822	0.818	0.812	0.816	0.825
H18	0.738	0.845	0.745	0.754	0.759	0.756	0.757	0.763	0.766
H19	0.734	0.776	0.710	0.707	0.710	0.712	0.713	0.723	0.716
H20	0.688	0.703	0.706	0.724	0.715	0.710	0.715	0.726	0.719
H21	0.748	0.783	0.760	0.780	0.777	0.772	0.763	0.776	0.773
H22	0.712	0.805	0.701	0.732	0.711	0.710	0.713	0.724	0.721
H23	0.869	0.965	0.847	0.853	0.874	0.873	0.852	0.851	0.872
H24	0.734	0.802	0.734	0.763	0.744	0.746	0.751	0.766	0.761
H25	0.727	0.811	0.712	0.712	0.725	0.724	0.703	0.723	0.713
H26	0.730	0.787	0.737	0.747	0.746	0.745	0.744	0.755	0.751
H27	0.713	0.785	0.764	0.774	0.774	0.776	0.778	0.792	0.788
H28	0.748	0.931	0.792	0.812	0.817	0.812	0.790	0.814	0.807
H29	0.700	0.742	0.702	0.733	0.710	0.711	0.719	0.741	0.728
H30	0.799	0.868	0.776	0.774	0.791	0.791	0.773	0.775	0.785
H31	0.751	0.793	0.796	0.818	0.815	0.818	0.807	0.813	0.824
H32	0.806	0.820	0.772	0.781	0.789	0.790	0.783	0.802	0.797
H33	0.714	0.811	0.746	0.760	0.759	0.760	0.749	0.771	0.761
H34	0.709	0.718	0.718	0.741	0.727	0.728	0.733	0.756	0.742
H35	0.736	0.782	0.724	0.733	0.735	0.733	0.730	0.741	0.738
H36	0.778	0.808	0.749	0.767	0.767	0.763	0.758	0.778	0.770
H37	0.742	0.836	0.728	0.738	0.739	0.739	0.741	0.753	0.750

Table S21 (continued) Valuation of health states adjusted for non-traders, in comparison with fitted values from the seven types of regression models

Health state	Observed TTO		Fitted utilities						
	mean	median	linear	beta	cens.	ordin.	scaled linear	scaled beta	scaled cens.
H38	0.711	0.796	0.740	0.767	0.748	0.747	0.754	0.765	0.760
H39	0.713	0.642	0.719	0.721	0.723	0.727	0.736	0.758	0.742
H40	0.725	0.787	0.708	0.724	0.713	0.713	0.717	0.737	0.722
H41	0.752	0.931	0.764	0.793	0.788	0.784	0.762	0.784	0.779
H42	0.668	0.677	0.685	0.683	0.694	0.691	0.684	0.704	0.689
H43	0.787	0.862	0.740	0.767	0.748	0.747	0.754	0.765	0.760
H44	0.735	0.723	0.732	0.753	0.744	0.746	0.745	0.771	0.756
H45	0.746	0.810	0.730	0.747	0.741	0.740	0.750	0.766	0.759
H46	0.838	0.880	0.755	0.759	0.765	0.765	0.761	0.761	0.769
H47	0.737	0.782	0.753	0.758	0.769	0.769	0.759	0.785	0.772
H48	0.752	0.810	0.802	0.818	0.822	0.818	0.812	0.816	0.825
H49	0.742	0.766	0.765	0.775	0.784	0.783	0.765	0.786	0.779
H50	0.756	0.846	0.746	0.758	0.754	0.756	0.751	0.769	0.760
H51	0.746	0.790	0.747	0.761	0.761	0.761	0.749	0.778	0.761
H52	0.739	0.796	0.734	0.760	0.751	0.745	0.739	0.767	0.749
H53	0.689	0.777	0.728	0.741	0.734	0.738	0.741	0.766	0.750
H54	0.779	0.839	0.769	0.798	0.785	0.784	0.783	0.790	0.795
H55	0.736	0.797	0.712	0.737	0.721	0.721	0.718	0.751	0.726
H56	0.688	0.735	0.710	0.704	0.714	0.715	0.719	0.728	0.723
H57	0.772	0.797	0.770	0.782	0.787	0.786	0.770	0.779	0.783
H58	0.738	0.703	0.695	0.707	0.700	0.702	0.711	0.738	0.718
H59	0.741	0.823	0.741	0.761	0.750	0.749	0.755	0.764	0.762
H60	0.767	0.806	0.769	0.798	0.785	0.784	0.783	0.790	0.795
H61	0.722	0.717	0.752	0.779	0.765	0.767	0.765	0.763	0.777
H62	0.729	0.780	0.749	0.770	0.763	0.762	0.758	0.764	0.768
H63	0.732	0.852	0.736	0.733	0.742	0.745	0.748	0.759	0.755
H64	0.820	0.861	0.799	0.812	0.816	0.814	0.802	0.812	0.814
H65	0.888	0.961	0.847	0.853	0.874	0.873	0.852	0.851	0.872
H66	0.846	0.926	0.847	0.853	0.874	0.873	0.852	0.851	0.872
H67	0.835	0.936	0.847	0.853	0.874	0.873	0.852	0.851	0.872
H68	0.847	0.936	0.847	0.853	0.874	0.873	0.852	0.851	0.872
H69	0.855	0.947	0.828	0.840	0.854	0.851	0.831	0.836	0.850
H70	0.766	0.839	0.798	0.815	0.825	0.823	0.796	0.815	0.816
H71	0.802	0.845	0.828	0.840	0.854	0.851	0.831	0.836	0.850
H72	0.855	0.901	0.809	0.827	0.831	0.828	0.814	0.825	0.830
H73	0.575	0.534	0.579	0.552	0.572	0.575	0.582	0.645	0.581
